# Elevated FOXG1 in glioblastoma stem cells cooperates with Wnt/β-catenin to induce exit from quiescence

**DOI:** 10.1016/j.celrep.2023.112561

**Published:** 2023-05-26

**Authors:** Faye L. Robertson, Eoghan O’Duibhir, Ester Gangoso, Raul Bardini Bressan, Harry Bulstrode, Maria-Ángeles Marqués-Torrejón, Kirsty M. Ferguson, Carla Blin, Vivien Grant, Neza Alfazema, Gillian M. Morrison, Steven M. Pollard

**Affiliations:** 1 Centre for Regenerative Medicine & Edinburgh Cancer Research UK Centre, Institute for Regeneration and Repair, University of Edinburgh, Edinburgh EH16 4UU, UK

## Abstract

Glioblastoma (GBM) stem cells (GSCs) display phenotypic and molecular features reminiscent of normal neural stem cells and exhibit a spectrum of cell cycle states (dormant, quiescent, proliferative). However, mechanisms controlling the transition from quiescence to proliferation in both neural stem cells (NSCs) and GSCs are poorly understood. Elevated expression of the forebrain transcription factor FOXG1 is often observed in GBMs. Here, using small-molecule modulators and genetic perturbations, we identify a synergistic interaction between FOXG1 and Wnt/β-catenin signaling. Increased FOXG1 enhances Wnt-driven transcriptional targets, enabling highly efficient cell cycle re-entry from quiescence; however, neither FOXG1 nor Wnt is essential in rapidly proliferating cells. We demonstrate that FOXG1 overexpression supports gliomagenesis *in vivo* and that additional β-catenin induction drives accelerated tumor growth. These data indicate that elevated FOXG1 cooperates with Wnt signaling to support the transition from quiescence to proliferation in GSCs.

## Graphical abstract

**Graphical abstract:**
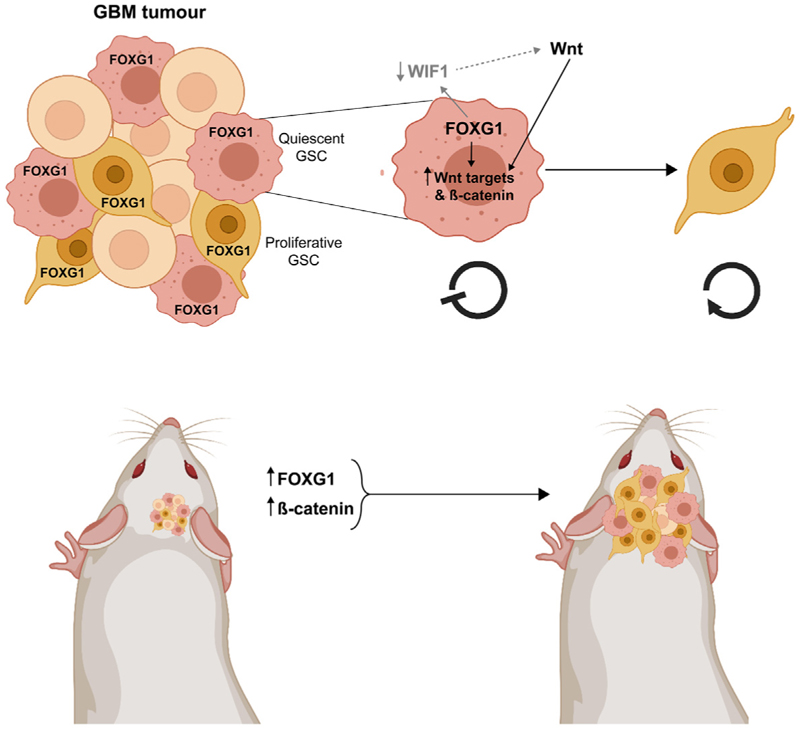

## Introduction

Glioblastomas (GBMs) are aggressive, incurable primary brain tumors with a median survival of just over a year.^1,2^ Almost all patients suffer fatal relapse following re-growth of the tumor after standard therapies (surgical debulking, chemoradiotherapy, and adjuvant chemotherapy). GBMs display inter-tumoral and intra-tumoral heterogeneity at many levels, including genetic drivers, epigenetic landscapes (heritable changes in gene expression resulting from mechanisms independent of changes to the genome sequence), and transcriptional circuits.^3,4^ However, while genetically heterogeneous, GBMs invariably contain cells with neural stem cell (NSC) identity that are thought to drive tumor growth. GBM stem cells typically express key NSC molecular markers, including neurodevelopmental transcription factors such as *SOX2, SOX9, FOXG1*, and *POU3F2*. These genes are functionally important in supporting the unconstrained self-renewal that underpins tumor growth.^5–8^

GBM stem cells (GSCs) are also heterogeneous in terms of their cell cycle state.^3^ Pathologists who score mitotic figures and Ki67 (MIB1) immunoreactivity have noted this for decades. However, with the advent of single-cell transcriptomics, it has become clear that a significant fraction of the tumor cell population exists in a quiescent (slow-cycling) or even dormant (non-cycling) state^3^ and that this cell fraction is enriched in tumorigenic cells.^9^ Thus, not all cells within the tumor are functionally equivalent in terms of their proliferative output. This heterogeneity must be considered alongside genetic, epigenetic, and transcriptional heterogeneity,^10^ as distinct cell states will likely have different roles in supporting tumor growth and evolution. Current therapeutic strategies focus on targeting actively proliferating GBM cells. However, following surgical debulking, residual cells in the resection margin have quiescent stem cell properties.^11^ Given the relative resistance of these populations to chemo- and radio-therapy,^12,13^ it is perhaps unsurprising that post-chemo-/radiotherapy residual tumor cell populations are enriched in quiescent GSCs. These quiescent tumor cells then drive regrowth of the tumor and patient relapse. This has been elegantly demonstrated using genetically engineered mouse models of GBM.^14^

Recent advances in our understanding of normal adult NSC quiescence regulation can help guide the exploration of quiescence control in GBM.^15^ Equally, regulators of GBM cell cycle control will likely mirror mechanisms used in normal NSC biology. However, the specific molecular genes and pathways controlling exit from the NSC quiescent state and how these are disrupted in GBM remain unclear. This knowledge will be needed to develop therapeutic approaches rationally designed to eliminate both quiescent and proliferative GSCs.

Bone morphogenetic protein (BMP) and epidermal growth factor (EGF) pathways promote NSC quiescence and proliferation, respectively, controlling the balance between cell states.^16–18^ We and others have shown that elevated FOXG1 contributes to gliomagenesis by attenuating the ability of BMP and related signals to trigger quiescence—in part by suppressing FOXO3 at transcriptional and post-transcriptional levels.^19,20^ This suggests that FOXG1, which we have shown is not required for GSC proliferation,^19^ is an important regulator of cell cycle re-entry from quiescence. Moreover, GSCs derived from patient tumors consistently show elevated expression levels of FOXG1.^21,22^ Elevated FOXG1 is, however, insufficient to support efficient exit from quiescence, and the majority of quiescent NSCs engineered to overexpress FOXG1 remain unresponsive to mitogens. Other pathways may therefore restrict competence for mitogen responsiveness and cell cycle re-entry.

Here, to search for pathways that may cooperate with FOXG1 in regulating quiescence exit, we performed a screen of pharmacological small molecules in NSCs. This led us to uncover a striking synergy between elevated FOXG1 and Wnt signaling. The role of Wnt in GBM has been nebulous. Mutations in components of the Wnt signaling pathway are not significant drivers of GBM and do not trigger glioma formation when mutated in mouse models.^23^ NSCs *in vitro* can be expanded in the absence of exogenous Wnt (using just EGF and FGF-2), yet Wnt receptors and ligands are clearly expressed in the adult NSC niche and in GBM tumors, suggesting some functional role.^24–27^ Several studies have identified a role for Wnt in modulating tumor stem cell state, and, consistently, Wnt pathway activity correlates with poorer patient outcomes.^26,28–30^

Our findings suggest that Wnt has a specific role in quiescent GSCs, regulating the exit from quiescence in cooperation with FOXG1. However, when cells are fully proliferative in response to EGF/FGF, Wnt/FOXG1 is dispensable. Wnt/β-catenin signaling, therefore, has distinct functional roles depending on the cell cycle status of the cell. This explains why Wnt pathway activity is neither required to sustain proliferative NSCs *in vitro* nor frequently selected for mutation in GBM. Altogether, our findings suggest that Wnt inhibitors may have value in preventing the reawakening of quiescent GSCs.

## Results

### A small-molecule screen uncovers a GSK3 inhibitor that supports NSC exit from quiescence in the context of FOXG1/SOX2 induction

We previously reported an *in vitro* model system to explore NSC quiescence: BMP4 drives proliferating mouse NSCs into a quiescent NSC/astrocyte-like state that is largely unresponsive to the mitogens EGF/FGF-2^19^; upon induction of FOXG1 and SOX2, a subset of these cells can become responsive to EGF/FGF-2, re-enter the cell cycle, and re-express markers of radial glialike NSCs (e.g., *Nestin, Fabp7, Sox2*, and *Olig2*). However, cell cycle re-entry in this model is not efficient. Most NSCs remain unresponsive and post-mitotic—either dormant or differentiated. We reasoned that there must be other pathways underlying competence for mitogen responsiveness and efficient cell cycle re-entry. Indeed, we previously reported that knockout of the cell cycle repressor FOXO3 synergizes with FOX/SOX to drive cell cycle re-entry. Here, we used a chemical screen of known pharmacological modulators of key stem cell and cancer pathways to search for additional limiting pathways (StemSelect Library).

**Figure 1:**
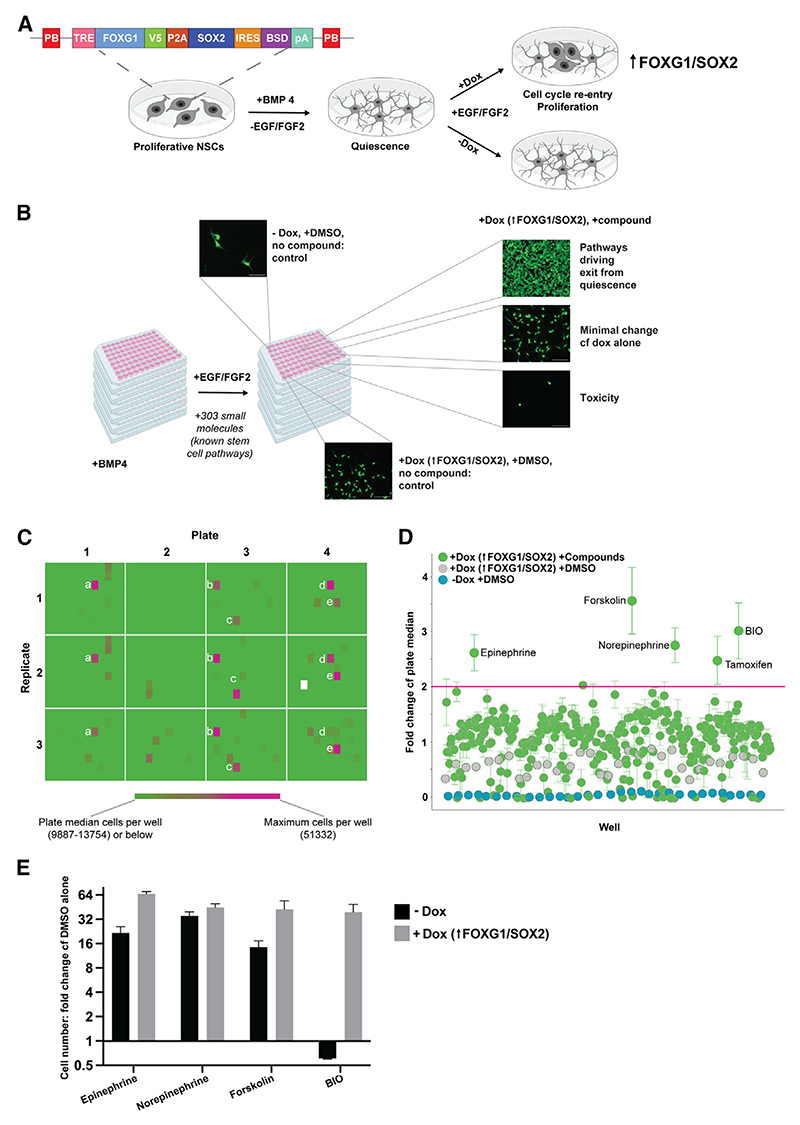
GSK3 inhibition enables efficient cell cycle re-entry in the context of FOXG1/SOX2 overexpression (A and B) Schematic of the screening process. Cells with conditional FOXG1/SOX2 over-expression and Foxo3 deletion are driven out of cycle by BMP4 treatment. Compounds inducing cell cycle re-entry in the presence of FOXG1/SOX2 induction are screened by assessment of cell number and morphology at 6 days. Created with BioRender. (C) Heatmap of cell number per well at screen endpoint, across 4 plates in triplicate, showing 5 potential hits. A scale showing increased cell number above plate median is adopted to highlight wells with a clearly high cell number. a = epinephrine, b = forskolin, c = norepinephrine, d = tamoxifen, and e = BIO. Controls are in columns 1 and 12 and have cell number below the plate median. (D) Scatterplot of cell count expressed as fold change cf. plate median. Means of 3 replicates ± SD are shown. Red line indicates threshold for calling hits, 2 × plate median. Tamoxifen did not validate as a hit (see also Figures S1D and S1E). (E) Validation of the 4 hits ± Dox; FC in cell number per well cf. EGF+FGF2+DMSO control. Performed in triplicate. Mean ± SEM. The concentration of compounds varied depending on the library concentration: all compounds were used at 1:10,000. Refer also to Figure S1. PB, PiggyBac inverted terminal repeat; TRE, tetracycline response element; V5, V5 protein tag; P2A, 2A self-cleaving peptide; IRES, internal ribosome entry site; BSD, blasticidin S deaminase; pA, polyadenylation site; NSCs, neural stem cells; BMP4, bone morphogenetic protein 4; EGF, epidermal growth factor; FGF-2, fibroblast growth factor 2; DMSO, dimethylsulfoxide.

For this cell-based phenotypic screen, we used a previously reported transgenic NSC cell line (FOD3), which harbors a TET-inducible FOXG1-2A-SOX2 expression cassette combined with *Foxo3* knockout (Figure 1A). These cells, therefore, model a key feature of GSCs, namely the excessive levels of FOXG1 and SOX2. The Z′ for the screening assay reached 0.6 for FOD3 cells, while it was lower for cells with intact *Foxo3* (Figure S1H) (Table S1). The FOD3 cell line, therefore, provided an optimal cellular model for compound screening. Cells were plated at low density in BMP4 media for 24 h and then exposed to EGF/ FGF-2 mitogens with doxycycline (Dox) plus library compounds in 96-well format (n = 3; Figure 1B). Culture plates were fixed 7 days after the addition of compounds. Nuclei were stained with DAPI for quantification, alongside HCS CellMask staining to monitor morphological changes (flat spread astrocytic-like appearances, with multiple processes, in quiescence, to bipolar proliferating NSCs; Figures 1B and S1A). Significant hits were defined as compounds that induced cell cycle re-entry with >2-fold increase in cell number (mean nuclei count) over plate median (Figures 1C and 1D).

Four validated hits were identified; three related to the cAMP pathway (epinephrine, norepinephrine, forskolin). The fourth hit, the glycogen synthase kinase-3 (GSK3) inhibitor 6-bromoin-dirubin-3′-oxime (BIO) suggested that Wnt signaling might be a critical cooperating pathway of either FOXG1, SOX2, or FOXO3. Importantly, we tested the effects of these four hits without Dox and found that only BIO triggered proliferation solely in the context of FOXG1/SOX2 overexpression (with Dox) (Figures 1E, S1B, and S1C). These data suggested a potential synergistic interaction between GSK3 inhibition and FOXG1/SOX2 overexpression, supporting highly efficient exit from the quiescent NSC state.

### Two distinct GSK inhibitors, BIO and Chiron, cooperate with FOXG1 to stimulate the proliferation of quiescent NSCs

**Figure 2:**
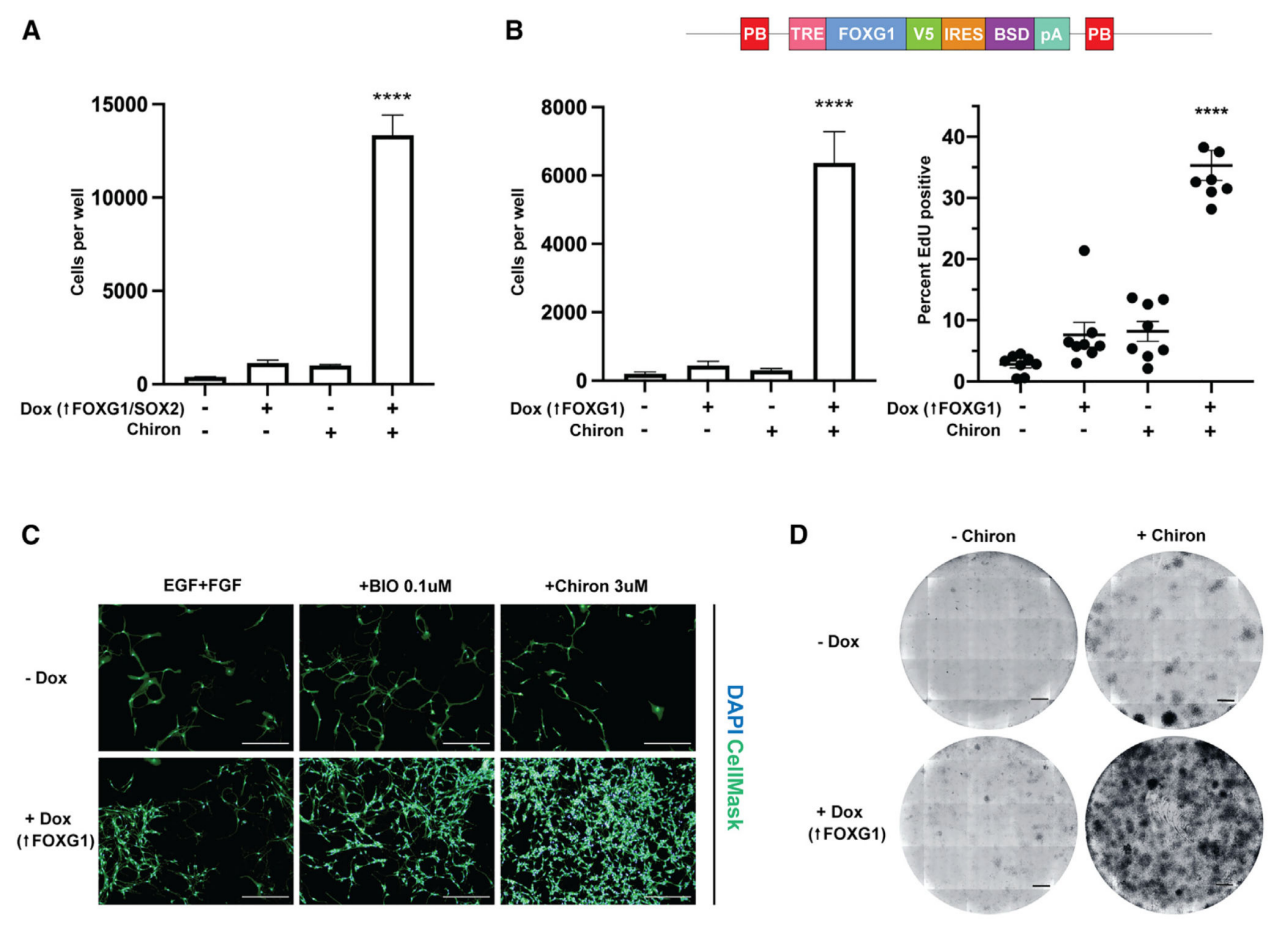
Highly efficient exit from quiescence into a proliferative state can be achieved by elevating FOXG1 levels and inhibiting GSK3 (A) Increase in FOD3 cell number after BMP4 exposure for 24 h and return to EGF+FGF2 ± Dox and/or Chiron for 6 days. One-way ANOVA with Dunnett’s multiple comparison tests. n = 3. Significance shown for comparison with EGF+FGF2. (B) Dox-inducible human FOXG1-V5 cassette. Quantification of cell number in F6 cells (inducible FOXG1 overexpression) in the same assay. One-way ANOVA with Dunnett’s multiple comparison tests. n = 5 independent replicates, >3 technical replicates each. EdU incorporation in F6 cells by condition. Friedman test. n = 8 independent replicates, 15 technical replicates each. Significance shown for comparison with EGF+FGF2. Equivalent assay in cells with inducible SOX2 alone is shown in Figures S1F and S1G. Mean ± SEM. (C) Representative images of F6 cells in the same assay (+/– Dox and/or BIO or Chiron). HCS CellMask (green), DAPI (blue). Scale bars, 150 μm. (D) Representative images of colony-forming assays: F6 cells plated at 5,000 cells/well (6 well plate, 5 cells/mm^2^) in BMP4 for 24 h or EGF+FGF2 ± Dox/Chiron for 10 days. Scale bars, 2 mm. Refer also to Figure S2.

We next determined if CHIR99021 (Chiron), an alternative GSK3 inhibitor with increased potency and selectivity, would give similar results to BIO. Indeed, Chiron was extremely effective at triggering cell cycle re-entry when delivered with FOXG1-SOX2 induction (Figure 2A). Furthermore, given the genetic interactions previously reported between FOXG1 and Wnt during development, we hypothesized that FOXG1, rather than SOX2, or Foxo3 loss, synergizes with GSK3 inhibition.^31,32^ Indeed, in cells with FOXG1 induction alone, but not SOX2, and with intact *Foxo3* (FOXG1-V5 only; F6 cells), we also saw highly efficient exit from quiescence (Figures 2B and 2C). ~35% of cells in the plus Dox and Chiron condition were driven into the cell cycle, based on EdU incorporation (2 h pulse), compared with <5% of cells in EGF+FGF alone (Figure 2B). Using colony-formation assays, we also confirmed the synergistic effects of FOXG1 induction with Chiron (Figure 2D). A high proportion of cells re-entered the cell cycle and generated colonies from their previously quiescent state (Figure 2D). To evaluate the efficiency of colony formation more accurately, cells were plated in serial dilution: 10,000, 1,000, and 100 cells, respectively. The higher two concentrations each led to a confluent plate at 7–10 days, and 100 cells yielded ~30 colonies (Figure S2E). This ~30% efficiency of colony formation is exceptionally high, especially given that the colony-forming efficiency of proliferative NSCs is typically ~10% (Figure S2F). We conclude that a synergistic effect of elevated FOXG1 and GSK3 inhibition stimulates highly efficient cell cycle re-entry of quiescent NSCs. To confirm that colonies that formed upon exposure to Dox+Chiron had NSC properties, we stained them for Nestin and performed serial passage colony assays in EGF+FGF2 media, demonstrating colony formation after two passages (Figure S2G). Additionally, we differentiated colonies formed after Dox+Chiron exposure with astrocytic and neuronal differentiation protocols (Figures S2H and S2I). We have previously shown that BMP4 induces a dormant quiescent state, whereas BMP4+FGF2 induces a primed quiescent state.^18^ The effect of FOXG1+GSK3 inhibition upon exit from quiescence is evident in either model (Figures 2B, and S2C). We found that 24 h BMP4 exposure induces a shallower quiescence than 72 h BMP4 exposure, similar to 72 h BMP4+FGF2 (Figures S2A and S2B).

### Wnt/β-catenin signaling pathway synergizes with FOXG1 overexpression to enable efficient exit from quiescence in NSCs

**Figure 3:**
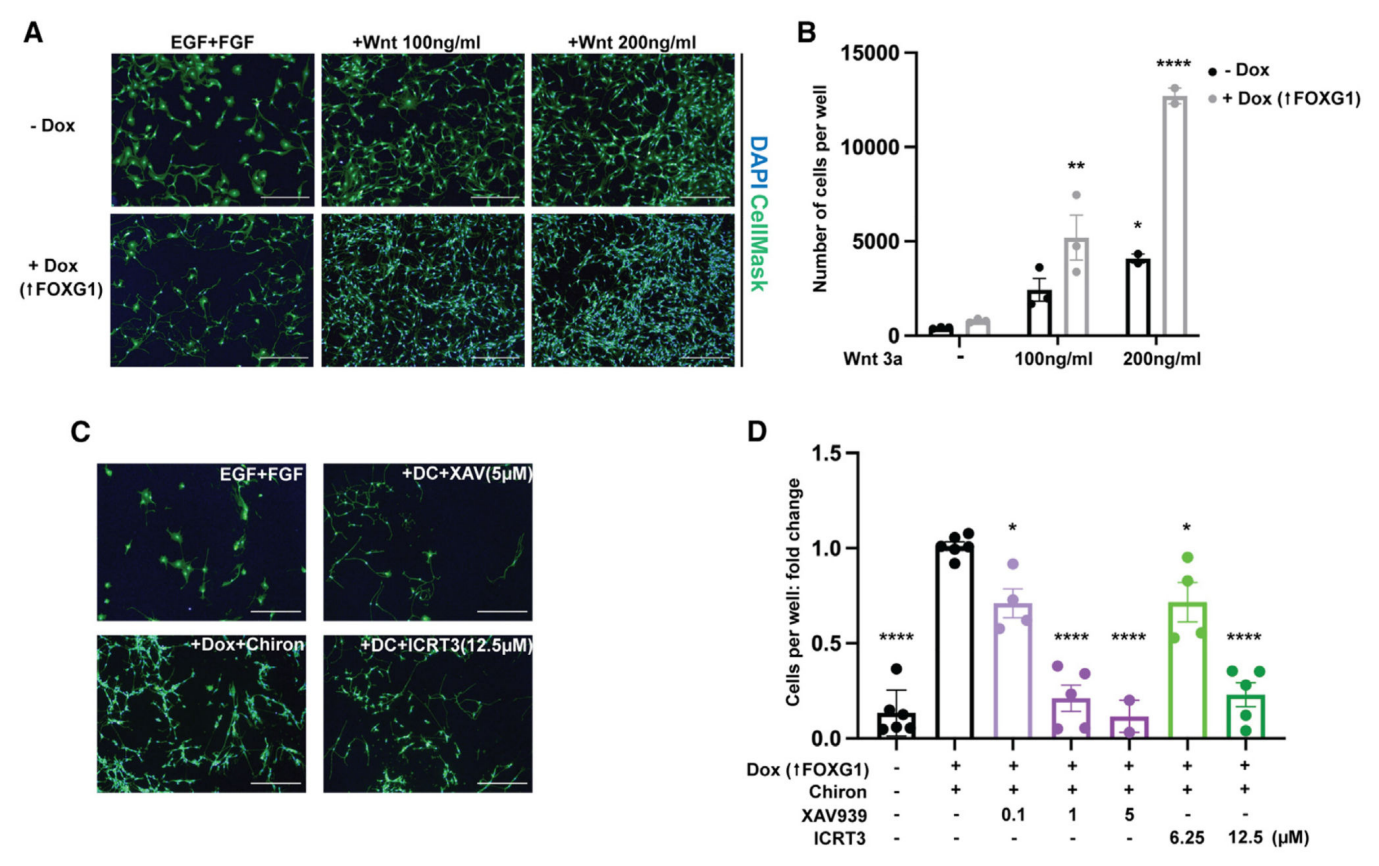
Elevated Wnt activity synergizes with FOXG1 in driving cell cycle re-entry (A) Representative images of F6 cells following exposure to BMP4 and return to EGF+FGF2 ± Dox and/or Wnt3a. HCS CellMask (green), DAPI (blue). (B) Quantification of cell number per well at assay endpoint. Two-way ANOVA. n = 3 independent replicates, 6 technical replicates each. Significance shown for comparison with EGF+FGF2. Mean ± SEM. (C) Representative images of F6 cells in the BMP4/return to EGF+FGF2 assay in the presence or absence of Wnt inhibitors XAV939 and ICRT3. HCS CellMask (green), DAPI (blue). Scale bars, 150 μm. DC, +Dox+Chiron. (D) Both Wnt inhibitors resulted in a significant reduction in cell number in the cell cycle re-entry assay. One-way ANOVA. n = 6 independent replicates, >5 technical replicates each. Significance shown for comparison with Dox+Chiron condition. Mean ± SEM. Refer also to Figure S3.

GSK3 is part of the β-catenin destruction complex and its inhibition leads to increased stability of β-catenin, the key downstream effector of the canonical Wnt signaling pathway. However, GSK3 has also been reported to modulate many other signaling pathways, including Notch, Hedgehog, and others.^33^ To determine if the effects we observed with Chiron are primarily due to activation of the Wnt signaling pathway, we first tested if exogenous Wnt ligands could phenocopy Chiron. Indeed, quantitative analysis of proliferation confirmed that Wnt3a, in the context of FOXG1 induction (plus Dox), could trigger a similar efficiency of cell cycle re-entry and cell morphological changes to Chiron (Figures 3A and 3B).

We next tested if two different pharmacological inhibitors of Wnt signaling could abrogate the effect of the FOXG1(Dox)+Chiron synergy: XAV939 is a tankyrase inhibitor that stabilizes axin, antagonizing Wnt signaling,^34^ and ICRT3 is a specific inhibitor of β-catenin-responsive transcription in the nucleus, downstream of GSK3.^35^ Blockade of the Wnt signaling pathway eliminated the synergistic effects of FOXG1 and Chiron in triggering cell cycle re-entry of NSCs in the quiescent state without significant cell death (Figures 3C, 3D, and S3B). Of note, neither exposure to Wnt3a ligand nor to the Wnt inhibitors at these doses affected proliferation of actively cycling NSCs (Figure S3A), suggesting that Wnt signaling has a specific role in the exit from quiescence. The above data confirm that FOXG1 and the Wnt signaling pathway cooperate to enable efficient exit from quiescence in NSCs during the initial transition from a quiescent to a cycling state.

**Figure 4:**
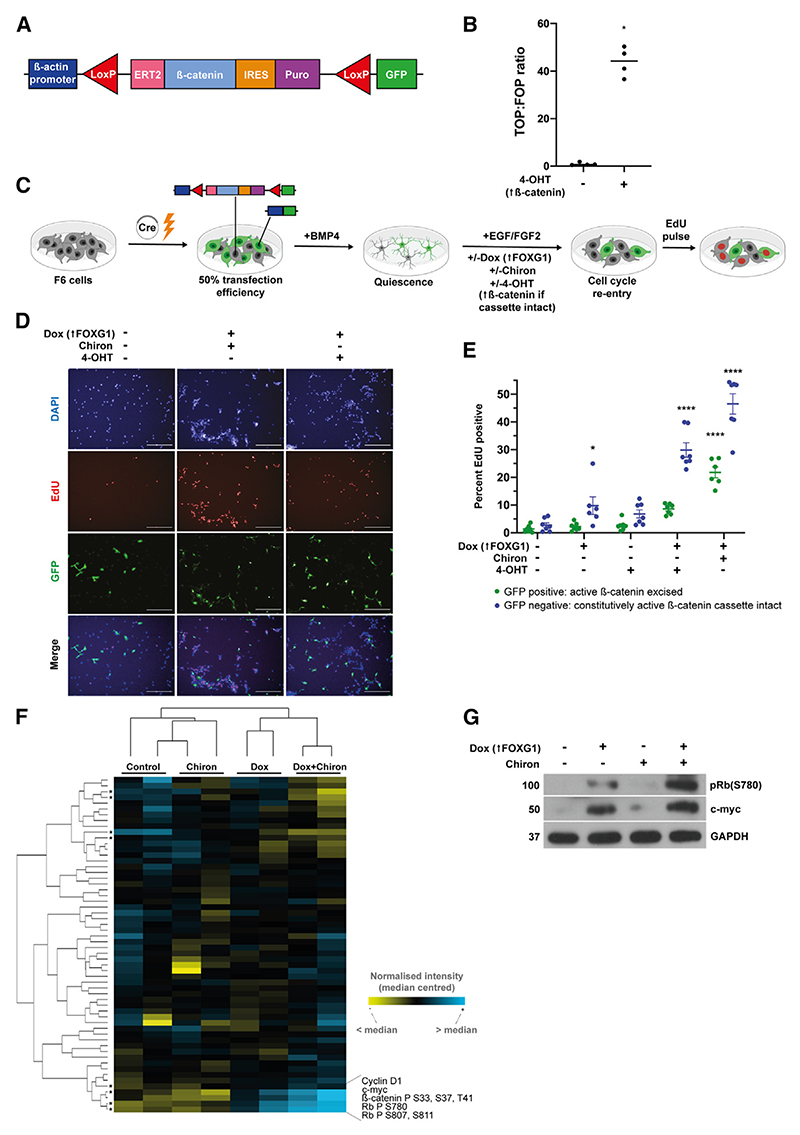
Highly efficient exit from quiescence into a proliferating state can be achieved by elevating FOXG1 levels and β-catenin activity FOXG1 elevation leads to Wnt target gene activation. (A) Schematic of the tamoxifen-inducible constitutively active β-catenin cassette. (B) TOPflash assay confirms Wnt pathway activation in F6BC1 cells after exposure to the active metabolite of tamoxifen, 4-hydroxytamoxifen (4-OHT), for 48 h. n = 4 independent replicates, 6 technical replicates each. Two-tailed Mann Whit-ney test. (C) Schematic of the assay to assess synergy between FOXG1 and β-catenin in exit from quiescence. Cre-mediated excision is ~50% efficient, resulting in a mixed population of cells with an intact cassette (and therefore GFP^−^) and cells in which the cassette has been excised (GFP^+^). These populations are expected to have a differential response to 4-OHT. Created with BioRender. (D) Representative images of F6BC1 cells, transfected with Cre-expression plasmid to excise the β-catenin cassette in a subpopulation of cells, after BMP4 exposure and return to EGF+FGF2 for 4 days. GFP (green), DAPI (blue), EdU (red). (E) EdU incorporation by condition showing that cells with the cassette excised (GFP^+^) retain response to Dox+Chiron but not Dox+4-OHT; cells with the cassette (GFP^−^) exit quiescence with Dox + either Chiron (GSK3 inhibition) or 4-OHT (induction of β-catenin). Two-way ANOVA with Sidak’s multiple comparison tests. n = 7 independent replicates, >5 technical replicates each. 4-OHT 1 μM. Significance shown for comparison with –Dox–Chiron–4OHT. Mean ± SEM. Nuclei were delineated and scored using Columbus software algorithms and verified visually. (F) Hierarchical clustering of RPPA data show that F6 cells exposed to Dox, rather than Chiron, cluster with cells exposed to Dox+Chiron. The top upregulated products are Wnt targets or products of Wnt target activity. Asterisks mark proteins demonstrating a significant difference between the EGF+FGF2 and Dox+Chiron conditions. t tests with Holm-Sidak correction. Independent duplicates, technical triplicates. Additional significant proteins are listed in Figure S4C. (G) Confirmatory western blot for top hits. GAPDH is used as a loading control. Refer also to Figure S4. *LoxP*, locus of x-over P1; ERT2, tamoxifen-inducible estrogen receptor ligand binding domain; IRES, internal ribosome entry site; Puro, puromycin resistance sequence; GFP, green fluorescent protein.

We next took a genetic approach to further confirm that the canonical β-catenin pathway lies downstream of Chiron and Wnt3a. We used a previously reported tamoxifen-inducible-(ERT2)-β-catenin cassette. With this approach, the addition of 4-hydroxytamoxifen (4-OHT) results in nuclear translocation of N-terminally truncated, stabilized, constitutively active β-catenin; removal of the cassette, via Cre-mediated excision, is reported by activation of GFP expression (Figure 4A).^36,37^ Using plasmid nucleofection, this cassette was stably integrated into F6 (FOXG1-inducible) NSCs. Clonal lines (Dox-inducible FOXG1 plus 4-OHT-inducible β-catenin; hereafter termed F6BC1 cells) were derived and validated. We confirmed that these F6BC1 NSCs respond to 4-OHT, with inducible Wnt activation, using the TOPflash luciferase transcriptional reporter assay (Figure 4B). Cells were treated transiently with Cre to generate a mixture of ~50% 4-OHT-inducible β-catenin cells (GFP^−^, cassette intact) and non-inducible (GFP^+^, cassette excised) cells. This provides an internal negative reference control allowing the investigation of cell-autonomous effects. After BMP4-induced quiescence, cells were returned to mitogens in the presence of Dox and/or Chiron, 4-OHT, or both. We scored the percentage of proliferation in the GFP^+^ and GFP^−^ populations (Figure 4C). This experimental system confirmed that β-catenin induction, in GFP^−^ cells, phenocopies the effects of Chiron in stimulating cell cycle re-entry and proliferation (Figures 4D and 4E). GFP^+^ cells, lacking the 4-OHT-inducible β-catenin cassette, did not re-enter the cycle efficiently with Dox+4-OHT, suggesting that the effects of FOXG1/β-catenin are cell autonomous (i.e., there is no rescue of cell cycle entry in adjacent 4-OHT-unresponsive cells). As expected, GFP^+^ cells remained responsive to Dox+Chiron. We conclude that there is a cooperation between Wnt/β-catenin signaling and elevated FOXG1 that is sufficient (in the presence of EGF/FGF2) to induce cell cycle re-entry of quiescent NSCs.

### Enhanced expression of Wnt target genes is observed in the presence of elevated FOXG1

To interrogate the potential mechanism of the synergy, we initially screened for differences in protein expression of key signaling pathways using reverse phase protein array (RPPA). A time point of 3 days after return to EGF/FGF-2 was selected to capture changes occurring prior to the majority of cells re-entering the cell cycle. At this time point, we found only minimal increases in the mitotic marker phospho-Plk1 (Figure S4B). FOXG1 was upregulated to similar levels by Dox and Dox+-Chiron, as determined by qRT-PCR, confirming that the effect of the addition of Chiron is not due to anomalous further upregulation of FOXG1 (Figure S5A). Hierarchical clustering showed that cells treated with Dox (FOXG1 upregulation) clustered with those treated with the combination of Dox+Chiron. In contrast, cells treated with Chiron clustered with those returned to EGF+FGF alone (Figure 4F), indicating that FOXG1 is the driver of many of the differences in protein expression. The proteins significantly downregulated and upregulated in the Dox+Chiron conditions are shown in Figure S4C. Of note, the four proteins significantly upregulated included known Wnt target genes, c-Myc and cyclin D1^38,39^, as well as two phospho-Rb proteins downstream of cyclin D1 (Figures 4F and S4C). c-Myc and phos-pho-Rb upregulation was confirmed by western blot (Figure 4G; quantified in Figures S4F and S4G), with a consistent pattern of upregulation by FOXG1, further increased with Chiron, with some upregulation also observed using Chiron alone. Although these are well-established Wnt targets, they are not exclusively regulated by the Wnt pathway. Accordingly, we conducted western blotting for Axin2 to confirm the upregulation of Wnt activity by Dox, by Chiron and, further, by Dox+Chiron (Figures S4D and E).

**Figure 5:**
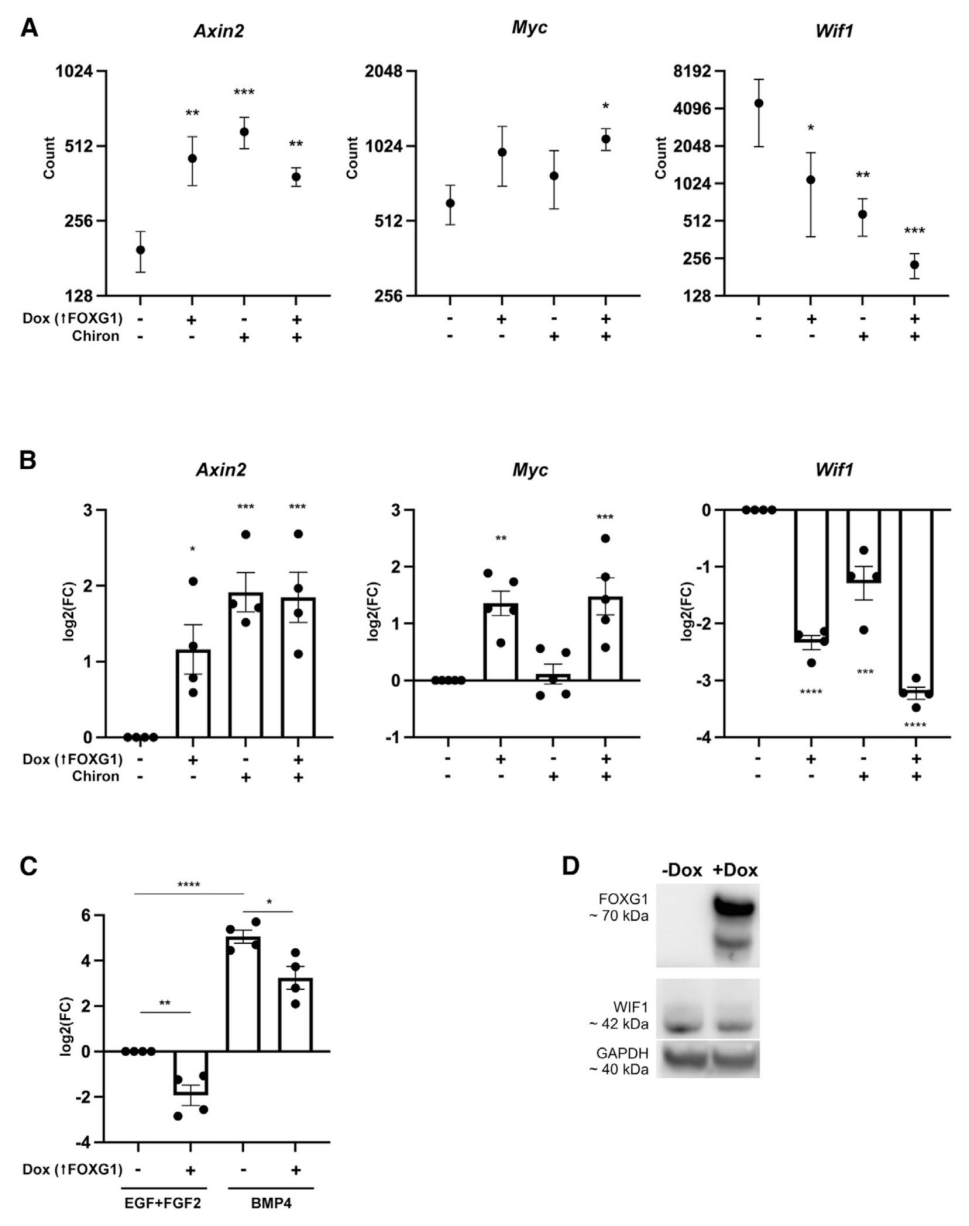
FOXG1 elevation leads to Wnt target gene modulation and repression of Wif1 (A) The most differentially expressed gene in the NanoString dataset is the Wnt inhibitor *Wif1*, which is downregulated in Dox, Chiron, and Dox+Chiron compared with EGF+FGF2 alone. NanoString was conducted on RNA extracted from F6 cells after 72 h in BMP+FGF2 followed by 48 h in EGF+FGF2 ± Dox and/or Chiron. Significance shown is for comparison with EGF+FGF2 alone. Performed in independent triplicate. A full list of genes is shown in Table S2. (B) qRT-PCR for Wnt target gene expression in F6 cells after 72 h in BMP+FGF2 followed by 72 h in EGF+FGF2 ± Dox and/or Chiron. One-way ANOVA, n = 4 independent replicates, 3 technical replicates. Significance shown is for comparison with EGF+FGF2 alone. (C) qRT-PCR analysis of *Wif1* mRNA levels in F6 cells cultured in BMP4 ± Dox or EGF+FGF2 ± Dox for 24 h. n = 4 independent replicates, technical duplicates. Two-tailed t test. (B and C) Expression values were normalized to *Gapdh* and shown relative to the expression in EGF+FGF-2 –Dox (in which log2(FC) = 0). The y axis represents log2(FC), equivalent to –ddCt value. All graphs show mean ± SEM. (D) Western blot analysis of WIF1 expression in F6 cells ± Dox for 24 h grown in NS cell media (EGF/FGF). GAPDH is used as a loading control. Refer also to Figure S5.

The above data and known roles of Wnt and FOXG1 in other contexts led us to hypothesize that this synergistic pathway involves cell-autonomous changes to transcriptional programs. We therefore used the NanoString mRNA profiling technology to assess the transcriptional levels of key markers associated with known hallmarks of cancer and cancer signaling pathways. This confirmed that transcription of *Myc* and *Axin2* was upregulated by Dox and Chiron, consistent with Wnt pathway activation (Figures 5A and 5B).

Also noteworthy, we uncovered *Wif1*, a well-established secreted Wnt signaling antagonist,^40^ as the most significantly repressed gene in the presence of Dox and Chiron (Figure 5). We confirmed that WIF1 protein levels are reduced by elevated FOXG1 (Figure 5D). This reduction in WIF1 levels in the context of FOXG1 could be expected to prime cells to respond to Wnt signaling and becomes functionally relevant at the exit from quiescence. This is consistent with our observation that Dox administration during BMP4 exposure primes cells to exit quiescence in response to Chiron, even where Dox is withdrawn (Figure S5B). Altogether, these observations indicate that elevated FOXG1 enables highly efficient activation of Wnt signaling that supports both transcriptional activation of β-catenin target genes and repression of negative regulators of the Wnt signaling pathway. This is consistent with a working model in which elevated FOXG1 in quiescent NSCs increases their responsiveness to Wnt/β-catenin signaling.

### FOXG1 and Wnt cooperate to support tumor progression in a murine model of glioblastoma

**Figure 6:**
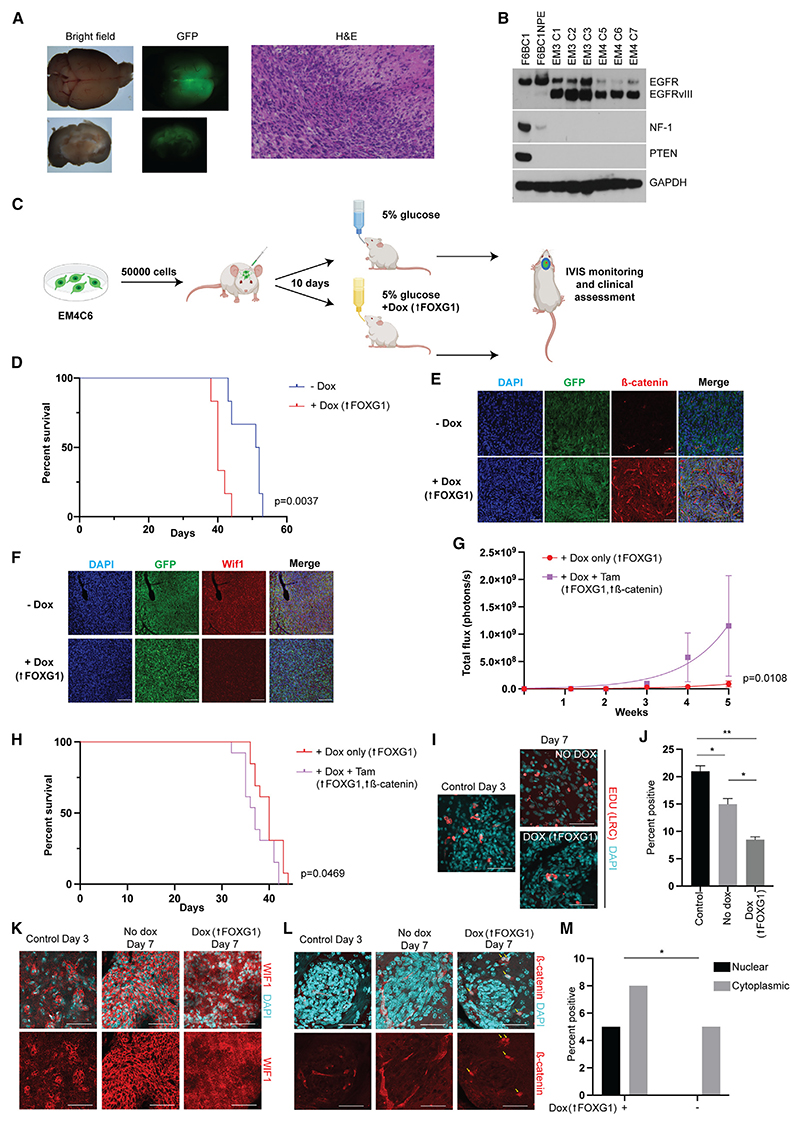
FOXG1 upregulation in GBM *in vivo*/*ex vivo* leads to increased β-catenin, reduced WIF1, reduced quiescent cell fraction, and shorter survival Additional induction of β-catenin leads to accelerated tumor growth and further reduces survival time. (A) GFP-expressing tumors form after orthotopic transplantation of F6BC1NPE cells. Hematoxylin and eosin (H&E) staining confirms GBM histology. (B) Western blot of clonal cell lines generated from tumors, showing gain of EGFRvIII and loss of NF-1 and PTEN, partial in the bulk population pre-transplantation and complete in the clonal lines derived after transplantation. GAPDH is used as a loading control. EM3 and EM4 refer to the mice from which tumors were taken and the clonal lines derived. (C) Schematic of *in vivo* experiment. F6BC1NPE cells (EM4 clone 6) were transplanted orthotopically into the brains of NSG mice. After 10 days, mice were given 2 mg/mL Dox in 5% glucose, or 5% glucose alone, as drinking water. IVIS imaging was conducted weekly. Created with BioRender. (D) Survival curve showing significant reduction in survival for mice given Dox. Log rank (Mantel Cox) test. n = 6 per group. (E) Representative immunohistochemistry images from tumors of mice given Dox or no Dox, showing increased β-catenin expression in the Dox condition. DAPI (blue), GFP (green), β-catenin (red). Scale bars, 50 μm. See also Figure S6E. (F) Representative immunohistochemistry images from tumors of mice given Dox or no Dox and culled at 21 days, showing reduced WIF1 expression in the Dox condition. DAPI, blue. GFP, green. WIF1, red. Scale bars, 50 μm. Quantified in Figure S6H. (G) Quantification of IVIS signal over time in two groups of mice given either Dox alone or Dox+i.p. tamoxifen (see also Figure S6C and D), showing faster growth in the Dox+tamoxifen group compared with Dox alone. Non-linear regression analysis, n = 9 per group. As the mice with the largest tumors in the Dox+tamoxifen (Tam) group had been culled by 5 weeks, last recorded values are carried over (from 3 or 4 weeks, widening the SEM but allowing comparison between groups). Mean ± SEM. (H) Survival curve showing reduction in survival for mice given Dox+Tam compared with Dox alone. Log rank (Mantel Cox) test. n = 13 per group. (I) Representative images from the engrafted tumor regions in organotypic slice culture exposed to EGF+FGF2 for 3 days, to allow engraftment, then EGF+FGF2 ± Dox for a further 4 days, showing label-retaining cells. EdU (LRCs, label-retaining cells; red); DAPI (blue). Scale bars, 50 μm. (J) Quantification of LRC fraction in the slice culture assay. One-way ANOVA with Tukey’s multiple comparison tests. Performed in duplicate. Mean ± SEM. (K) Immunohistochemistry for WIF1 shows increased signal and cytoplasmic staining after 7 days in EGF+FGF2 alone cf. 3 days. This is lost in the presence of Dox (FOXG1 overexpression). WIF1 (red), DAPI (blue). Scale bars, 50 μm. (L) In the presence of Dox, a proportion of cells express nuclear β-catenin (indicated by arrows) in keeping with Wnt pathway activation. β-catenin (red), DAPI (blue). Scale bars, 50 μm. (M) Quantification of proportion of cells in organotypic slice culture expressing nuclear or cytoplasmic β-catenin. Performed in duplicate and measured over 6 fields at 40 ×. Refer also to Figure S6.

We recently reported that GBM driver mutations (*EGFRvIII, Nf1* loss, and *Pten* loss) could be efficiently engineered into adult mouse NSCs using CRISPR-Cas9 technology plus PiggyBac transgenesis.^41^ The resulting cell lines efficiently induce GBM-like tumors following orthotopic transplantation.^41^ Using this strategy, we transformed the F6BC1 cells into a GBM-initiating cell model wherein we can exogenously control both FOXG1 levels and β-catenin (see STAR Methods). Tumor formation of F6BC1-NPE cells was confirmed by GFP imaging of freshly dissected whole brains and H&E staining (Figure 6A). We isolated the tumor mass and derived clonal cell lines, confirming that these had the triple combination of GBM driver mutations, tamoxifen-inducible β-catenin, and Dox-inducible FOXG1-V5 (Figures 6B and S6A). These cell lines were tumor initiating upon secondary transplantation into the striatum of a fresh cohort of mice.

Using this *in vivo* GBM model system, we were able to test the effects of FOXG1 and β-catenin on tumorigenesis. At day 10 following cell transplantation, half of the mice were given 2 mg/mL Dox in drinking water. Tumor formation was monitored using the IVIS luciferase imaging system (Figure 6C). FOXG1 induction in mice treated with Dox was confirmed by staining for the V5 epitope tag (Figure S6A). Importantly, elevated levels of FOXG1 significantly reduced survival (Figure 6D) and increased proliferation markers (Figures S6F and S6G). This finding is consistent with previously reported patient data showing that high FOXG1 expression is associated with poorer survival outcomes,^6^ as well as our experimental observations that FOXG1 is required for tumor growth in xenotransplantation GBM models.^19^

We next investigated the effect of the combination of FOXG1 upregulation with active Wnt/β-catenin induction by intraperitoneal (i.p.) injection of tamoxifen. Increased tumor growth (Figures 6G and S6D) and reduced survival time (Figure 6H) were seen. These data are consistent with our working model that elevated FOXG1 enables enhanced responsiveness to Wnt ligands from the tumor microenvironment *in vivo* and that β-catenin can phenocopy these effects. It is likely, however, that β-catenin induction by tamoxifen adds little to the already high levels of Wnt activity in the tumor microenvironment. β-Catenin expression is seen throughout the mouse brain (Figure S6B).^42^ With FOXG1 induction alone, elevated β-catenin expression is seen in the tumors, including nuclear β-catenin expression at earlier time points (Figures 6E and S6E), and this is consistent with findings in human GBMs, which express FOXG1 at high levels and show evidence of Wnt pathway activation.^26,28–30^ Additionally, Wif1 expression is reduced in the context of FOXG1 induction, consistent with our *in vitro* findings (Figures 6F and S6H). This supports our hypothesis that FOXG1 sensitizes cells to Wnt activation; further activation of the pathway accelerates tumor growth and reduces survival. In the context of the *in vitro* findings presented here, we suggest that this may result from increased exit from quiescence by tumor stem cells.

Exit from quiescence was anticipated to be an early event on exposure of cells to high FOXG1 and Wnt signaling, which is challenging to monitor following *in vivo* brain transplantation. Therefore, to interrogate these early events, we used an organotypic *ex vivo* brain slice culture assay to investigate the responses of engrafted tumor cells to FOXG1 overexpression.^43^ Tumorigenic cells were labeled with a 4 h EdU pulse before transplantation into brain slices within the striatum. The EdU signal is depleted and ultimately lost following rounds of mitotic divisions, so proliferating cells will lose EdU, whereas quiescent cells will retain the label. After 3 days, following engraftment into the slice, we exposed cells to Dox (FOXG1 overexpression) and compared them with controls with no Dox. On day 7, we found that cells exposed to Dox contained fewer EdU-positive, label-retaining (quiescent) cells than those cultured in mitogens without Dox (Figures 6I and 6J). Those exposed to Dox demonstrated lower levels of WIF1 expression and the presence of some cells with nuclear β-catenin expression, which was not seen in the slices with no Dox (Figures 6K–6M). These findings also indicate that cells with elevated FOXG1 are primed to re-enter the cell cycle and hence contribute to aggressive tumor growth, in keeping with *in vitro* findings (Figure S5B).

### The synergy between FOXG1 and GSK3 inhibition is relevant to human patient-derived glioblastoma stem cell lines

**Figure 7:**
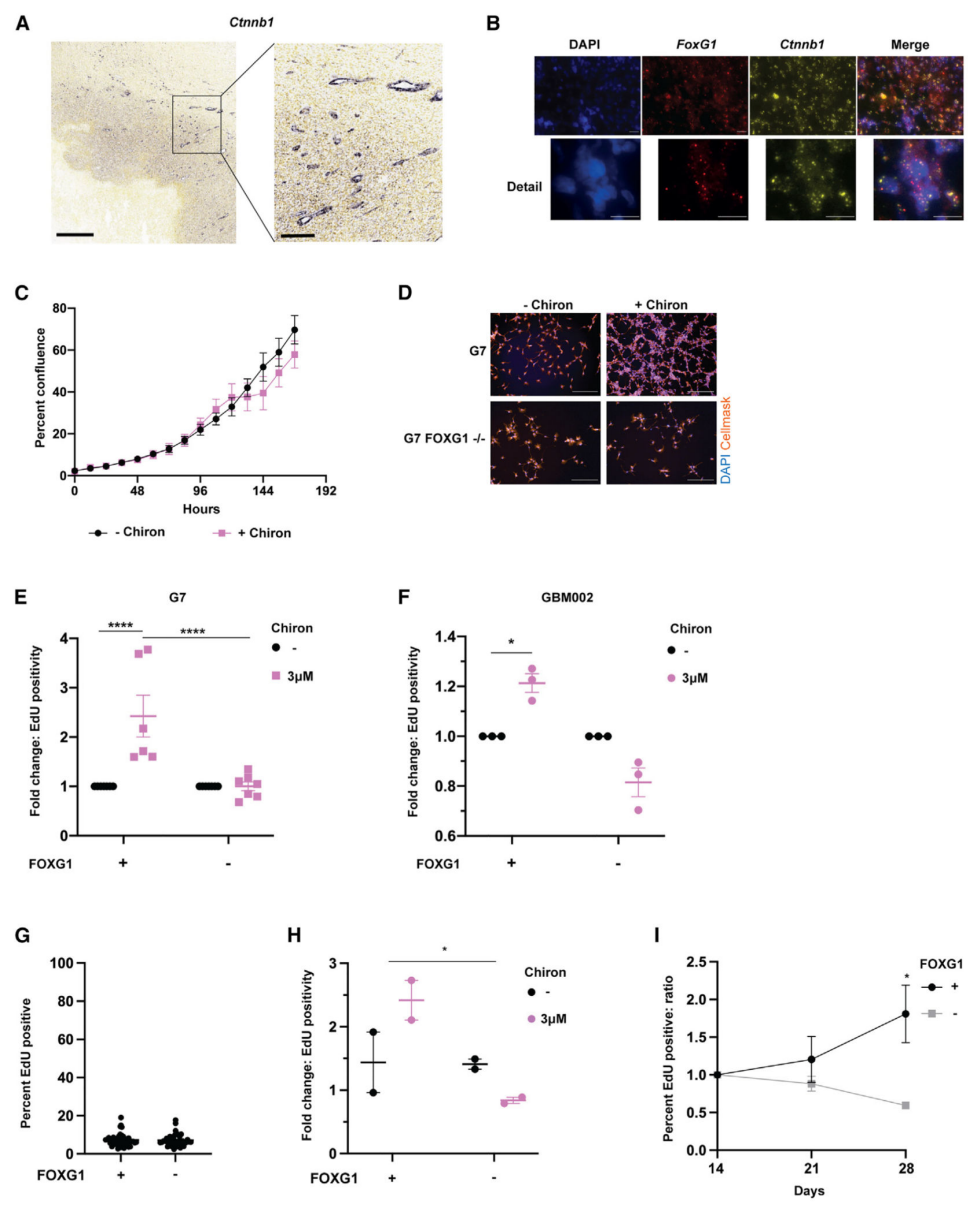
The synergy between FOXG1 and Wnt signaling is relevant to two human glioma cell lines (A) *In situ* hybridization for Ctnnb1 in an adult GBM specimen (Ivy GBM Atlas Project): *Ctnnb1* is expressed in tumor tissue, most markedly in perivascular regions. Scale bars, 800 and 200 μm (expanded image). (B) Representative images of RNAScope performed on human GBM tissue (G313). DAPI (blue), *FOXG1* mRNA (red), *CTNNB1* (β-catenin) mRNA (yellow). Scale bars, 50 μm. (C) Growth curve for G7 cells in EGF+FGF2 ± Chiron. 3 μM. n = 3. (D) Representative images of G7 cells after BMP-induced quiescence (8 days) and return to mitogens for 4 days showing response to Chiron only where FOXG1 is intact. DAPI (blue), HCS CellMask (orange). Scale bars, 150 μm. (E and F) Quantification of EdU incorporation in G7 and G7 FOXG1 KO cells (E) and in GBM002 and GBM002 KO cells (F), expressed as FC cf. EGF+FGF2 alone, showing that Chiron drives exit from quiescence in a dose-dependent manner only in the context of intact FOXG1. Two-way ANOVA with Sidak’s multiple comparison tests. (E) n = 6 independent replicates, >6 technical replicates each. (F) n = 3 independent replicates, 15 technical replicates. (G) EdU incorporation in GBM002 and GBM002 FOXG1KO cells 14 days after irradiation with 8 Gy n = 42 technical replicates in 2 independent experiments. (H and I) Impact of Chiron on EdU incorporation in GBM002 and GBM002KO cells. Cells, plated at 30 cells/mm^2^, were irradiated with 8 Gy at day 0. At day 14, either Chiron or DMSO was added to media. At day 21 or 28, a 2 h EdU pulse was performed. (H) FC in EdU incorporation at 28 days, compared with 14 days, in Chiron or DMSO control for the parental and FOXG1-KO cell lines. Data are expressed as FC to account for slight variations in baseline EdU and between two independent experiments. Two-way ANOVA, p = 0.0499. (I) Data expressed as ratio of EdU incorporation in Chiron:DMSO over time. Linear regression analysis, p = 0.0118. Graphs show mean ± SEM. Refer also to Figure S7.

We predicted that findings from our mouse overexpression model would extend to the human GSC context and that a synergy would exist between high FOXG1 expression and GSK3 inhibition. Consistent with published evidence of Wnt activation in human GBM (Figure 7A)^26–30^ and FOXG1 overexpression in human GBM,^6,21^ we confirmed colocalization of FOXG1 and β-catenin expression by RNAScope in human GBM tissue isolated at debulking surgery (Figures 7B and S7D). We previously published evidence of cell cycle exit in the majority of human GSCs after 8 days of continuous exposure to BMP4.^44^ Accordingly, we treated two patient-derived cell lines, G7 (an adult GSC line) and GBM002 (a pediatric GSC line), along with their CRISPR-Cas9 FOXG1-knockout derivatives,^19,45^ with BMP4 for 8 days. These cells were then re-exposed to EGF+FGF2 ± Chiron to assess exit from quiescence. Notably, there was a minimal effect of Chiron on the growth of cells in proliferative conditions (Figure 7C). However, after exposure to BMP4, cells with intact FOXG1 treated with Chiron were significantly more likely to re-enter the cell cycle than those returned to EGF+FGF2 without Chiron. This effect was not seen in cells that had ablation of FOXG1 (Figures 7D–7F, S7A, and S7B). To assess the synergy in the context of an additional clinically relevant quiescence model, GBM002 cells and the corresponding FOXG1-knockout (KO) line were irradiated with 8 Gy, and an absence of proliferation was observed. EdU incorporation was assessed at 14 days and was below 10% in almost all wells (Figure 7G). At 14 days, media were supplemented with either Chiron or DMSO, and EdU incorporation was reassessed at days 21 and 28. EdU incorporation and cell number increased with Chiron only in the context of intact FOXG1 (Figures 7H, 7I, S7E, and S7F). Although further work is needed to characterize quiescent cells in this assay, cells were non-cycling/slowly cycling post-irradiation and, as in a BMP-induced quiescence assay, began proliferating in response to Chiron, only in the context of FOXG1 expression. Taken together, these data suggest that a synergy between FOXG1 and Wnt signaling may be relevant to the regulation of quiescence in human GSCs.

## Discussion

Quiescent GSCs are relatively chemo- and radioresistant, and their reactivation leads to tumor recurrence.^14,46^ BMP signaling has been shown to regulate quiescence in NSCs, including GSCs.^44,47^ Here, using a BMP4-based *in vitro* model of quiescence and unbiased chemical screening, we have been able to uncover a synergistic molecular pathway between FOXG1 and GSK3 inhibition that supports cell cycle re-entry of quiescent NSCs. Our inducible FOXG1 cell lines were designed to model GSC biology, as FOXG1 is typically overexpressed in GSCs relative to NSCs. We show that elevated FOXG1 supports glioma-genesis in our inducible *in vivo* transplantation model and that the FOXG1/Wnt pathway operates in this context. Finally, the synergistic effect of FOXG1 and GSK3 inhibition is operational in human patient-derived GSCs—both adult and pediatric—suggesting disease relevance.

The specific roles of Wnt signaling in NSCs and GBMs still need to be better understood. Our findings help resolve some seemingly contradictory literature by revealing that Wnt/β-catenin has a specific cell-context-dependent role in supporting quiescent NSCs/GSCs to re-enter the cell cycle. Our findings are consistent with the function of Wnt in other tissue stem cells, where it provides a locally restricted niche signal that supports the maintenance of stem cell identity.^48,49^ A recent study from the Dirks group has shown that Wnt levels are variable in GSCs and that Wnt/β-catenin, along with Notch, is essential for self-renewal in a subset of cells with a pro-neural signature.^50^ In the mouse brain, Austin et al. found that Wnt signaling was dispensable for normal NSC homeostasis but that β-catenin stimulation resulted in state-specific effects on NSCs.^51^ The roles of Wnt are therefore highly cell-context dependent—not only in terms of GBM subtype, as has been previously shown, but also, as we show here, in the balance between quiescence and proliferation. In future studies, spatial transcriptomics and lineage tracing could help resolve whether the FOXG1/Wnt synergy has niche-specific roles, for example, in the perivascular niche, where β-catenin is expressed at high levels and where quiescent GSCs are found.^52^

Using our recently established protocols for the transformation of adult NSCs, and subsequent *in vivo* transplantation, we could generate GBMs in which FOXG1 could be overex-pressed. This demonstrated that FOXG1 overexpression leads to accelerated tumor growth and decreased survival, with increased proliferative GSCs relative to quiescent GSCs. This is consistent with previous *in vitro* findings. FOXG1 leads to increased activation of endogenous Wnt signaling, and additional induction of active β-catenin further increases the rate of tumor growth and decreases survival. Future studies should focus on further elucidating the transcriptional mechanism and key downstream effectors of the synergy between FOXG1 and Wnt activity.

The negative impact of FOXG1 overexpression on survival in our mouse model is consistent with previous findings that high FOXG1 mRNA levels in human GBM samples predict poorer overall survival outcomes and is an exciting corollary to the findings that FOXG1 knockdown in orthotopic GBM transplantation models results in improved outcomes^6,53^ and that FOXG1 KO in patient-derived GSCs abolishes tumorigenesis.^19^ FOXG1 frequently acts as a transcriptional repressor, with evidence of both direct and indirect means of repression.^31,54,55^ It is known to regulate the response to transforming growth factor β (TGF-β) signaling, via its action on FoxO-Smad complexes and repression of *p21cip1*, conferring resistance to TGF-β-mediated cytostasis.^53^ Given that our data suggested a cell-autonomous mechanism underlying the synergy, it is likely that FOXG1 operates genome wide, supporting enhanced regulation of a large cohort of Wnt target genes. However, we also identified the secreted factor, WIF1, as a potential downstream effector that may contribute.

In conclusion, our data suggest that elevated FOXG1 may sensitize quiescent GSCs to local Wnt signaling, thereby priming subsequent proliferative responses to EGF and FGF signaling pathways (or other RTK pathways). This would explain why high levels of FOXG1 are under positive selection in many GBMs. Furthermore, this model of FOXG1 helps explain why neither FOXG1 nor Wnt signaling is necessary in proliferating GBM cells (as they are specifically required in the context of quiescence). Hence, we predict that suppression of the FOXG1/Wnt pathway would be a poor choice as a first-line therapeutic target in a clinical setting, as it would likely fail to suppress the major proliferative cell component of GBM tumors. Nevertheless, we speculate that the suppression of FOXG1 or the Wnt pathway could suppress the reactivation of the quiescent cells left behind following debulking surgery and chemo-/radiotherapy. Wnt inhibitors may therefore be helpful to prevent the recurrence of GBMs after standard first-line treatments that focus on the proliferative cells.

### Limitations of the study

Our reductionist *in vitro* studies provide striking evidence of a synergy between FOXG1 and Wnt signaling in regulating NSC quiescence. Our *in vivo* findings are more modest but likely reflect the high baseline of Wnt ligand *in vivo* within the tumor microenvironment. Here, we showed a clear effect of FOXG1 overexpression on survival and an additional modest impact of β-catenin activation. As the interaction between FOXG1 and β-catenin upon exit from quiescence is likely to be an early event in a population for which few markers are validated, it is challenging to interrogate the synergy at a mechanistic level *in vivo*. Future studies could employ functional *in vivo* experiments with lineage tracing and single-cell profiling for detailed assessment of self-renewal and quiescence *in vivo*. As suggested above, spatial transcriptomics are likely to be helpful in identifying niche-specific roles of Wnt and FOXG1 and overcome the limitation of our primarily *in vitro* study. Finally, further work should make use of a broader range of human cell lines to elucidate the precise molecular mechanism of the FOXG1/Wnt synergy, which we suggest may relate to the sequestration of TLE by the FOXG1 Groucho-binding domain.

## Star★Methods

### Key Resources Table

**Table:** 

REAGENT or RESOURCE	SOURCE	IDENTIFIER
Antibodies		
Actin	Santa Cruz	Cat#: sc-1616
Axin2	Abcam	Cat#: 109307
β-catenin	BD	Cat#: 610154
c-MYC	Abcam	Cat#: 32072
FOXG1	In house	N/a
GAPDH	ThermoFisher	Cat#: 6C5
GFAP	Biolegend	Cat#: 28294
GFP	Abcam	Cat#: 13970
Ki67	ThermoFisher	Cat#: MA5-14520
Nestin	Developmental Studies Hybridoma Bank	Cat#: rat-401
NF1	Santa Cruz	Cat#: sc-67
Phospho-EGFR Tyr1068	Cell Signaling Technology	Cat#: 3777
Phospho-Rb (S780)	Abcam	Cat#: 47763
PTEN	Cell Signaling Technology	Cat#: 9556
SOX2 (IF)	Millipore	Cat#: 5603
SOX2 (WB)	R&D	Cat#: MAB2018
TuJ1	Biolegend	Cat#: 801202
V5	eBioscience	Cat#: -6796-82
WIF1	Abcam	Cat#: 186845
Biological samples		
Glioma tissue and derived cells	Glioma Cellular Genetics Resource, CRUK, UK	http://gcgr.org.uk
Chemicals, peptides, and recombinant proteins		
DMEM/HAMS-F12	Sigma	Cat#: D8437
Pen/Strep	Gibco	Cat#:15140-122
Glucose	Sigma Aldrich	Cat#: G8644
MEM-NEAA (100X)	Gibco	Cat#: 11140-035
BSA Solution	Gibco	Cat#:15260-037
Beta Mercaptoethanol	Gibco	Cat#: 31350-010
B27 Supplement (50X)	LifeTech/Gibco	Cat#: 17504-044
N2 Supplement (100X)	LifeTech/Gibco	Cat#: 17502-048
Recombinant Mouse EGF	Peprotech	Cat#: 315-09
Recombinant Human FGF	Peprotech	Cat#: 100-18b
Laminin	Cultrex	Cat#: 3446-005-01
Accutase	Sigma Aldrich	Cat#: A6964
Glutamine	Gibco	Cat#: 25030-021
Mouse Recombinant BMP4	Peprotech	Cat#: 5020-BP
Potassium Chloride	Sigma Aldrich	Cat#: P3911
Methanol	Fisher Scientific	Cat#: 13298233
DAPI	Thistle Scientific	Cat#: 30-45-01
SG Cell Line Transfection Kit	Lonza	Cat#: V4XC-3032
Blasticidin	Invivogen	Cat#: ANT-BL-1
Hygromycin B	Life Technologies	Cat#: 10687010
DMSO	Sigma Aldrich	Cat#: 276855
dNTPs	Thermo Scientific	Cat#: R0191
LongAMPTaq Polymerase	NEB	Cat#: M0323
Paraformaldehyde Powder 95%	Sigma	Cat#: 158127
Triton X-100	Merck Life Sciences	Cat#: X-100
Goat Serum	Sigma Aldrich	Cat#: G6767
Milk Powder	Marvel	N/A
Tween 20	Cambridge Bioscience	Cat#: TW0020
SuperScript III	Invitrogen	Cat#: 18080093
Sodium Azide	Fisher Scientific	Cat#: 12615117
PBS Tablets	Sigma Aldrich	Cat#: P4417
Ethanol	VWR	Cat#: 20821-330
FluoroSave Reagent	Calbiochem	Cat#: 345789
DNase	Sigma Aldrich	Cat#: 101041590001
FBS	Gibco	Cat#: 10270-106
Taqman Universal PCR Master Mix	Applied Biosystems	Cat#: 4305719
D-Luciferin potassium salt	Cambridge Bioscience	Cat#: CAY14681
SuperG Blocking Buffer	Grace Bio Labs	Cat#: 105100
IRDye 800CW Streptavidin	LI-COR Biosciences	Cat#: 926-32230
cOmplete ULTRA protease inhibitor	Roche	Cat#: 05056489001
PhosSTOP phosphatase inhibitor	Roche	Cat#: 04906837001
HCS CellMask	ThermoFisher	Cat#: H32714, H32713
Recombinant mouse Wnt3a	R&D	Cat#: 1324-WN-002
CHIR99021	Axon Medchem	Cat#: 1386
Critical commercial assays		
RNeasy Mini Kit	Qiagen	Cat#: 74104
Click-iT EdU Cell Proliferation Kit for Imaging	ThermoFisher	Cat#: C10377
Click-iT TUNEL Alexa Fluor Imaging Assay	ThermoFisher	Cat#: C10245
Dual Luciferase Reporter Assay System	Promega	Cat#: E1910
Mouse Gapdh Taqman gene expression assay	ThermoFisher	Mm99999915_g1
Mouse Axin2 Taqman gene expression assay	ThermoFisher	Mm00443610_m1
Mouse Foxg1 Taqman gene expression assay	ThermoFisher	Mm02059886_s1
Human FOXG1 Taqman gene expression assay	ThermoFisher	Hs01850784_s1
Mouse Myc Taqman gene expression assay	ThermoFisher	Mm00487804_m1
Mouse Wif1 Taqman gene expression assay	ThermoFisher	Mm00442355_m1
RNAScope Multiplex Fluorescent Kit v2 Assay	ACD	N/A
nCounter PanCancer Pathways Panel	Nanostring	N/A
Deposited data		
Nanostring	This manuscript	Robertson, Faye; Pollard, Steven (2023), “Nanostring”, Mendeley Data, V1, https://doi.org/10.17632/v4k77m3rxm.1
Experimental models: Cell lines		
FOD3	(Bulstrode. H., et al.)^19^	N/A
S15	(Bulstrode. H., et al.)^19^	N/A
F6	(Bulstrode. H., et al.)^19^	N/A
F6BC1	This paper	N/A
F6BC1NPE and derived lines	This paper	N/A
pGBM002 (HSJD-GBM-002)	(Hennika, T., et al.)^56^	N/A
aGBM7 (G7)	(Pollard S. M., et al.)^57^	N/A
GCGR Human Glioma Stem Cells	This paper, Glioma Cellular Genetics Resource, CRUK, UK	N/A
Experimental models: Organisms/strains		
Mouse: NSG (NOD-scid-gamma)	Charles River (original source, colony bred in house)	Cat#: 614NSG
Oligonucleotides		
Refer to Table S3	This manuscript	N/A
Recombinant DNA		
Cas9D10A-2A-GFP	Addgene	Cat#: 44720
PiggyBAC transposase	Austin Smith, University of Cambridge	N/A
Gateway pDONR™221	ThermoFisher	Cat#: 12536017
M50 Super 8x TOPflash	Addgene	Cat#: 12456
M51 Super 8x FOPflash	Addgene	Cat#: 12457
ERT2-β-catenin	Austin Smith, University of Cambridge	N/A
Software and algorithms		
Incucyte® Base Software	Essen Bioscience	https://www.essenbioscience.com/en/products/incucyte/
Fiji/ImageJ	Open Source	https://imagej.net/Fiji
BioRender	BioRender	https://biorender.com/
IGV (version 2.8.2)	(Thorvaldsdottir, H., et al.)^58^	http://software.broadinstitute.org/software/igv/
GraphPad Prism 9.0	GraphPad Software, Inc	https://www.graphpad.com/
Spotfire	Tibco	https://www.tibco.com/products/tibco-spotfire
StratoMineR	Core Life Analytics	https://corelifeanalytics.com/
Columbus	PerkinElmer	https://www.perkinelmer.com/uk/product/image-data-storage-and-analysis-system-columbus
Harmony	PerkinElmer	https://www.perkinelmer.com/uk/product/harmony-4-9-office-license-hh17000010
Living Image Software v.4.5.2	PerkinElmer	https://resources.perkinelmer.com/corporate/content/lst_software_downloads/release-notes-li-4.5.2.pdf
Mapix	Innopsys	https://www.innopsys.com/product/corporate/mapix-software/
Cluster 3.0	Open Source	http://bonsai.hgc.jp/~mdehoon/software/cluster/
Java TreeView 3.0	Open Source	https://jtreeview.sourceforge.net/
nSolver 4.0	Nanostring	https://nanostring.com/products/analysis-solutions/nsolver-advanced-analysis-software/

### Resource Availability

#### Lead contact

Further information and requests for resources should be directed to, and will be fulfilled by, the lead contact, Steven Pollard (steven.pollard@ed.ac.uk)

#### Materials availability

All reagents generated in this study (including cell lines and plasmids) are available on request from S.M.P.

### Experimental Model and Study Participant Details

#### Mice and *in vivo* procedures

All animal work on NSG (NOD-SCID gamma; non-obese diabetic, severe combined immunodeficiency with null mutation in IL2Rγ) mice was performed in accordance with protocols approved by Home Office UK guidelines in a designated facility under a project license to S.M.P. (PC0395462) at the University of Edinburgh. Mice were maintained on a regular diet in a pathogen-free facility on a 12-h light/dark cycle with unlimited access to food and water. For NSCs transplants, 6–8 week old male mice were anesthetized with inhalation vapor mix of oxygen at 2 L/min and isofluorane (Zoetis UK Ltd: VM 42058/4195) at 4% for induction and at 2–3% for maintenance on a stereotaxic frame. Stereotactic coordinates used were 1.5mm lateral, 0.6mm anterior to the bregma and 2.5mm deep. NSCs concentrated (~5 × 10^4^/μL) were injected in a volume of 2μL with a Hamilton syringe at 0.2 μL/min. Mice were given doxycycline 2 mg/ml in drinking water with 5% glucose, or 5% glucose alone. Where tamoxifen was administered, mice were given intraperitoneal tamoxifen 120 mg/kg or sunflower oil vehicle on day 1 (after transplantation on day 0). Monitoring of tumor growth *in vivo* was conducted by bioluminescence imaging 20 min after D-Luciferin (potassium salt) subcutaneous injection (50 mg/kg, Cayman chemical) using the IVIS Lumina LT Series III (PerkinElmer) instrument. Bioluminescence signals were analyzed using Living Image Software v.4.5.2 (PerkinElmer). Animals culled due to symptoms or signs of deterioration were included in survival analysis.

### Method Details

#### Cell culture

NSCs were isolated from the adult SVZ and maintained *in vitro* in presence of EGF and FGF-2 and laminin. Cells were cultured at 37°C and 5% CO2 and grown on uncoated tissue culture plastic. Dissociation was performed using accutase (Sigma). Cells were passaged 1:6 to 1:8, or media changed as appropriate, every 3–4 days. For colony forming assays NSCs were plated at low density (5000 cells per well-6 multiwell plate, 5 cells/mm^2^) in BMP4 for 24 h or BMP4+FGF2 for 72 h, when media were changed to fresh self-renewal media. For 96 well plate assays, cells were plated at 1000 cells per well (30 cells/mm^2^). For the screen, plating was performed using the Multidrop Combi reagent dispenser (ThermoFisher 5840300); compound addition was performed using the CyBio Felix liquid handler (AnalytikJena). For induction of quiescence, BMP4 for 24 h or BMP4+FGF2 for 72 h were used and led to equivalent levels of EdU incorporation (Figures S2A and S2B). BMP4+FGF2 for 72 h was the predominant quiescence assay.^18^ Self-renewal media: Mouse and human neural stem NSCs and GSCs were grown under serum-free conditions in DMEM F-12 supplemented with N2 and B27, penicillin-streptomycin, 1 μg/mL Laminin, 10 ng/mL EGF and 10 ng/mL FGF.^57,59^ Selection media contained puromycin, hygromycin or blasticidin. BMP4 (Peprotech, AF-120-05ET-100), FGF2 (Peprotech, # 100-18b) EGF (Peprotech (#315-09). Astrocyte differentiation assay: 10% FCS for 5 days. Neuronal differentiation assay: withdrawal of EGF for 24hr followed by with-drawal of FGF-2 for 7 days. Growth curves were generated using an IncuCyte live-cell imaging system. DMSO was used as a control in assays where compounds were added to media, unless otherwise stated. Details of the G7 and GBM002 cell lines and FOXG1 knockout have previously been published.^19,45^

#### Cell transfection

Design and construction of CRISPR sgRNAs is described in.^60^ The Amaxa (Lonza) nucleofection system was used. The pulse programs used were X005 (human cells), T030 (mouse PiggyBac) and DN100 (mouse random integration and CRISPR). In each case 1–2 million cells were transfected. For inducible PiggyBac constructs, a total of 6-12μg DNA was used, comprising pBASE, pCAG-Tet3G (PTre3G promoter, Clontech) and pDEST-TetOn (from pCAG Tet-On 3G Transactivator, Clontech) vector in 1:1:2 ratios. For CRISPR targeting, guide RNAs (x2), targeting vector (where appropriate) and Cas9 nickase were transfected in a 1:1:1:2 ratio. For single transfection NPE transformation, 1.5 million cells were transfected with 4.2ug DNA comprising cas9-mCherryNF1guide sequence x2, PTENgRNA sequence x2, PB-PyCAG-EGFRviii, PB-CAG-GFP-LUC-Ires-Bsd, pCMV-hyPBase in a 1:1:1:1:1:1:1 ratio. For random integration, 2 million cells were transfected with 1ug of linearised plasmid DNA. The tamoxifen-inducible(ERT2)-β-catenin plasmid was a kind gift from the laboratory of Prof Austin Smith.

#### Immunocytochemistry

Cells were fixed with 4% paraformaldehyde for 10 min, incubated in blocking buffer (10% normal goat serum and 0.2% Triton X-100 in 0.1M phosphate buffer saline) for 30 min, and incubated overnight at 4°C with the indicated primary antibodies: FOXG1 (1:3, Pollard lab), GFP (1:1000, Abcam 13970), Sox2 (1:100, Millipore 5603), V5 tag (1:1000, eBioscience 14-6796-82), β-catenin (1:500, BD 610154), TuJ1 (1:250 Biolegend 801202), Nestin (1:10 Developmental Studies Hybridoma Bank), GFAP (1:100 Biolegend 28294).

After several washes with PBS, immunoreactivity was detected with the appropriate Alexa Fluor-conjugated (Life Technologies) secondary antibody (1:1000). Cells were counterstained with 4^0^,6^0^, -diamidino-2-phenylindole (DAPI) and mounted with Fluorsave (Calbiochem). HCS CellMask (ThermoFisher H32714 green, H32713 orange) was used as per manufacturers’ instructions. EdU detection Click-it Thermo Fisher C10337 and TUNEL assay Click-it Thermo Fisher C10245 kits were used. Images were taken and analyzed using Confocal (Leica SP8, 3 and 5 detectors), Nikon TiE, or the PerkinElmer Operetta high content imaging system (with Harmony and Columbus software for image analysis). Quantification of signal intensity or of nuclei count, GFP or EdU positivity was conducted using Columbus algorithms or ImageJ. For Columbus algorithms, nuclei were selected using pre-set software parameters, selected objects were then subjected to exclusion criteria based on size, roundness, signal intensity and contact with the edge of the imaged field. Cytoplasm was delineated using pre-set software parameters and objects in contact with the edge of the imaged field were excluded. Selected objects were visually verified in a minimum of 25% of imaged fields in a minimum of 10% of imaged wells in each plate.

#### Immunohistochemistry

Brains were fixed in 4% PFA overnight at 4°C, then rinsed several times with PBS and stored in PBS +0.05% sodium azide. For histopathology procedures, brains were transferred into 70% ethanol and then embedded in paraffin for processing. 10mm coronal slices were prepared for hematoxylin and eosin (H&E) staining. For immunohistochemistry of fixed brain tissue, 50μm vibratome slices were transferred into a 24-well plate. Slices were incubated at room temperature for 30 min in blocking solution (0.2% Triton X-100 and 3% Goat Serum). Primary antibodies were incubated overnight at 4C as follows: Ki-67 (1:100 Thermo Fisher MA5-14520), GFP (1:300 Abcam13970), β-catenin (1:500, BD 610154), WIF1 (1:500, Abcam 186845). After three washes with PBS, slices were incubated with appropriate Alexa Fluor secondary antibodies (1:1000, Life technologies) and DAPI (1:2000, Sigma D9542) for 2 h. Slices were washed three times and were mounted on a slide with FluoroSave™ Reagent (345789, Calbiochem). Slices were examined with a confocal microscope (Leica TCS SP8).

#### Slice co-culture assay

Young adult mouse brains (5–6 weeks old) were removed, sliced and cultured.^43^ Cells growing *in vitro* were deposited in the striatum of the brain slices. Label retaining cells were labeled using EdU and, after being deposited in organotypic slice culture, were exposed to EGF+FGF2 for 3 days then EGF+FGF2 +/− Dox for a further 4 days. Co-cultures were fixed with PFA 4% and stained with primary and secondary antibodies^43^ and EdU cell proliferation click it kit (ThermoFisher). Samples were examined with a confocal microscope (Leica TCS SP8). Quantification was conducted using ImageJ software.

#### Western Immunoblotting

Immunoblotting was performed using standard protocols. Antibodies were diluted in 5% milk powder inTBS Tween 20 0.1%, and protein detection was carried out with HRP-coupled secondary antibodies and X-ray films. The following primary antibodies were used Axin2 (1:1000, Abcam 109307), SOX2 (1:400, R&D MAB2018), Phospho-Rb (S780) (1:500, Abcam 47763), c-MYC (1:1000, Ab-cam 32072), FOXG1 (1:50, hybridoma clone 17B12, Pollard lab), WIF1 (1:500, Abcam 186845), NF1 (1:500; Santa Cruz sc-67), PTEN (1:1000; CST 9556), phospho-EGFR Tyr1068 (1:1000; CST 3777), GAPDH (1:40000; ThermoFisher, 6C5), Actin (1:1000; Santa Cruz sc-1616). Quantification of band signal and normalisation to GAPDH signal was performed using ImageJ software and Excel.

#### Topflash assay

Cells were transfected with a Renilla luciferase plasmid and either the TOPflash plasmid, containing the TCF/LEF-Firefly luciferase expression construct (7 copies of the TCF/LEF transcriptional activator site upstream of firefly luciferase, a gift from Randall Moon via Addgene) or the FOPflash control, in which the TCF/LEF sites are mutated and cannot be activated by β-catenin. Renilla activity was recorded as a control for transfection efficiency and results were normalised to Renilla activity prior to determining the ratio between TOPflash and FOPflash firefly luciferase activity. The Promega Dual Luciferase Assay was conducted according to manufacturers’ instructions.

#### Quantitative real-time RT-PCR

RNA was extracted using the RNeasy spin column kit (Qiagen), plus DNase treatment to eliminate gDNA. cDNA was generated with SuperScript III (Invitrogen), and quantitative RT-PCR was performed using Taqman Universal PCR Master Mix (Applied Biosystems). The following Taqman assays (Life Technologies) were used: Axin2 (Mm00443610_m1), FoxG1 (Mm02059886_s1), FOXG1 (Hs01850784_s1), Myc (Mm00487804_m1), Wif1 (Mm00442355_m1).

#### RPPA

Samples were prepared using Lysis Buffer: 1% Triton X-100,50 mM HEPES, pH 7.4, 150 mM NaCl, 1.5 mM MgCl2, 1 mM EGTA, 100 mM NaF, 10 mM Na pyrophosphate, 1 mM Na3VO4, 10% glycerol, supplemented with cOmplete ULTRA protease inhibitor and PhosSTOP phosphatase inhibitor cocktails (Roche), on ice. Sample Buffer: 40% Glycerol,8% SDS, 0.25 M Tris-HCL, pH 6.8. Before use, 2-mercaptoethanol was added at 1/10 of the volume. Clarified supernatants in biological triplicate were adjusted to 2 mg/mL concentration and printed onto nitrocellulose-coated slides (Grace Bio-Labs) in a dilution series (four serial 2-fold dilutions) in technical triplicate using an Aushon2470 arrayer (Aushon Biosystems). Slides were blocked, probed with validated primary anti-bodies and detected with DyLight 800-conjugated secondary anti-bodies (New England BioLabs). Slides were read using an InnoScan 710-IR scanner (Innopsys) and quantified using Mapix (Innopsys). Relative fluorescence intensities were normalized to respective FastGreen-stained spots (total protein), and data were computationally analyzed as previously described.^61^

#### Nanostring

RNA was extracted from cells, and gDNA eliminated, using the RNeasy Mini Kit (Qiagen, 74104). The Nanostring PanCancer Pathways panel was used. Hybridization, purification and imaging on the nCounter system were conducted in accordance with manufacturers’ protocols. Raw protein count data were processed by applying background thresholding and content normalization in NanoString nSolver 4.0.

#### Creation of the F6BC1NPE line

F6BC1 cells were transfected using the DN 100 program of a 4D nucleofection system (Lonza). 1.5 x 10^6^ cells were resuspended in 100μL of SG transfection solution (Lonza). We delivered, via a single transfection, CRISPR gRNAs for *Nf1* and *Pten* deletion (+mCherry reporter), alongside PiggyBac CAG-EGFRvIII (+ hygromycin selectable), GFP and firefly luciferase (+ blasticidin selectable), to F6BC1 cells. Cells were sorted for dual GFP and Cherry positivity and subsequently exposed to hygromycin (100 μg/mL hygromycin) for 5 days and blasticidin (5 μg/mL) for 6 days sequentially to recover fully transfected cells. These pool of cells (200K) were transplanted into the striatum of 6 x NSG mice and in five of these mice we were able to see aggressive tumors by IVIS bioluminescence imaging of live animals.

#### RNAScope

RNAScope was conducted on 5μm slices of FFPE tissue prepared from human GBM samples obtained at the time of primary surgery. The RNAScope Multiplex Fluorescent Kit v2 Assay (ACD) was conducted in accordance with manufacturer’s instructions. Slices were examined with an inverted fluorescence microscope (Leica). G313 was obtained at surgery by Mr Paul Brennan.

#### Cell irradiation

Cells were cultured in adherent monolayer and irradiated in a Gammacell 40 Exactor (Best Theratronics) or sham irradiated. When removed from the incubator, culture plates were sealed with Parafilm (Bemis) until returned.

### Quantification and Statistical Analysis

Screening data were analyzed, and data visualisations created, with Spotfire (Tibco) and StratoMineR (Core Life Analytics). RPPA data were assessed using Cluster 3.0 and Java TreeView 3.0 and graphs created in GraphPad Prism 8. nSolver software was used for Nanostring analysis. Statistical analyses were performed in GraphPad Prism 8. Biological replicates were considered as different passage numbers of the same cell line plated in independent experiments. Mean and SEM are plotted unless otherwise stated. Statistical tests used are indicated in the figure legends. p values are denoted as follows * <0.05, ** <0.01, *** <0.001, **** <0.0001, ns > 0.05.

## Supplementary Material

Supplemental information can be found online at https://doi.org/10.1016/j.celrep.2023.112561.

Supplemental information

## Data Availability

Nanostring data have been deposited at the the Mendeley Data repository. Robertson, Faye; Pollard, Steven (2023), “Nano-string”, Mendeley Data, V1, https://doi.org/10.17632/v4k77m3rxm.1. DOIs are listed in the key resources table. This paper does not report original code. Any additional information required to reanalyse the data reported in this paper is available from the lead contact on request.

## Highlights

- A small-molecule screen reveals synergy between FOXG1 and GSK3 inhibition on quiescence exit
- Genetic and pharmacological approaches confirm the role of Wnt in the synergy
- FOXG1 enhances Wnt-driven transcriptional targets and represses WIF1
- Elevated FOXG1 and β-catenin in a GBM model drive accelerated growth and reduced survival

## In brief

Glioblastomas contain quiescent stem cells expressing high levels of FOXG1. Robertson et al. identify a synergistic interaction between FOXG1 and Wnt/β-atenin signaling that drives exit from quiescence *in vitro* and accelerated tumor growth *in vivo*. They show that FOXG1 enhances Wnt transcriptional targets and represses the Wnt inhibitor WIF1.
