# Supplementary material for: Elevated FOXG1 in glioblastoma stem cells cooperates with Wnt/β-catenin to induce exit from quiescence
Source: Cell Rep. Author manuscript; Available in PMC 2026 Feb 19. (PMC7618753; doi:10.1016/j.celrep.2023.112561)
Supplement: Supplemental information [file EMS212248-supplement-Supplemental_information.zip › 1-s2.0-S2211124723005727-mmc1.pdf]

Cell Reports, Volume *42*

Supplemental information

Elevated FOXG1 in glioblastoma stem cells cooperates with Wnt/β-catenin to induce exit from quiescence

Faye L. Robertson, Eoghan O'Duibhir, Ester Gangoso, Raul Bardini Bressan, Harry Bulstrode, Maria-Ángeles Marqués-Torrejón, Kirsty M. Ferguson, Carla Blin, Vivien Grant, Neza Alfazema, Gillian M. Morrison, and Steven M. Pollard

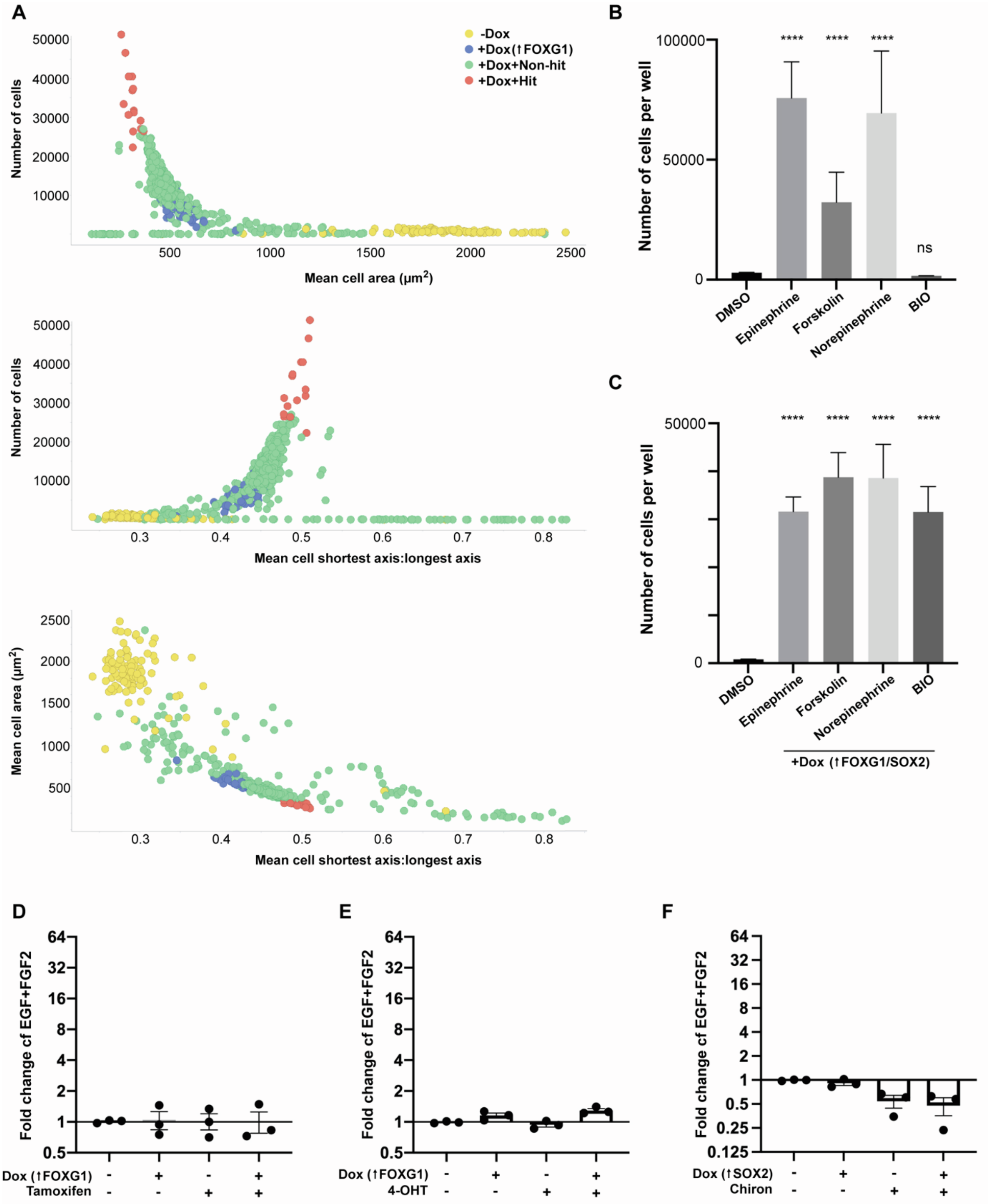

G

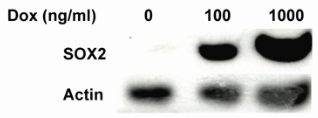

H

Mean +Dox	Mean -Dox	SD +Dox	SD -Dox	Z’
10477.27	1729.733	2629.409	435.2303	0.246832
8803.5	1858.767	2153.094	355.0453	0.294295
2021.067	717.6	452.8303	85.91881	0.409842
1969.967	726.9333	310.7397	61.65193	0.585756

Figure S1. Screening optimisation, validation and morphology data. Related to Figure 1. (A) Scatter plots of morphology data obtained from small molecule screen, showing distinct features of cells not exposed to Dox (larger, with long processes) and those exposed to Dox and those defined as hits. Refer also to Figure 1B, representative images. (B) Quantification of cell number for the 4 screen hits, plated in BMP4 and returned to EGF+FGF+DMSO or compound (no Dox). Performed in triplicate. One-way ANOVA with Dunnett's multiple comparison tests. Significance shown for comparison to EGF+FGF2+DMSO. (C) Quantification of cell number for the 4 screen hits, plated in BMP4 and returned to EGF+FGF+DMSO or compound (+Dox). Performed in triplicate. One-way ANOVA with Dunnett's multiple comparison tests. Significance shown for comparison to EGF+FGF2+DMSO. (D)&(E) Tamoxifen citrate and active metabolite 4-hydroxytamoxifen fail to drive cell cycle re-entry in repeat assays (BMP4 for 24 hours, return to EGF+FGF2 6 days +/- Dox and/or tamoxifen). Performed in independent triplicate; >10 technical replicates each. Scale to show comparison to validated hits (Figure 1E). Non-significant. One-way ANOVA. (F) SOX2 induction and GSK3 inhibition show no increase in exit from quiescence in the absence of FOXG1 induction. Fold change in S15 cell number (cf. EGF+FGF2 alone) by condition (EGF+FGF2 +/- Dox and/or Chiron). Non-significant. One-way ANOVA. Performed in independent triplicate; >10 technical replicates each. (G) Western blot confirming upregulation of human SOX2 by doxycycline in cells with Dox-inducible SOX2 only (S15 cells). Actin is used as a loading control. (H) Z' for 4 plates of FOD3 cells during screen optimisation. Each row represents a plate seeded using successive techniques and the Multidrop Combi reagent dispenser (ThermoFisher).

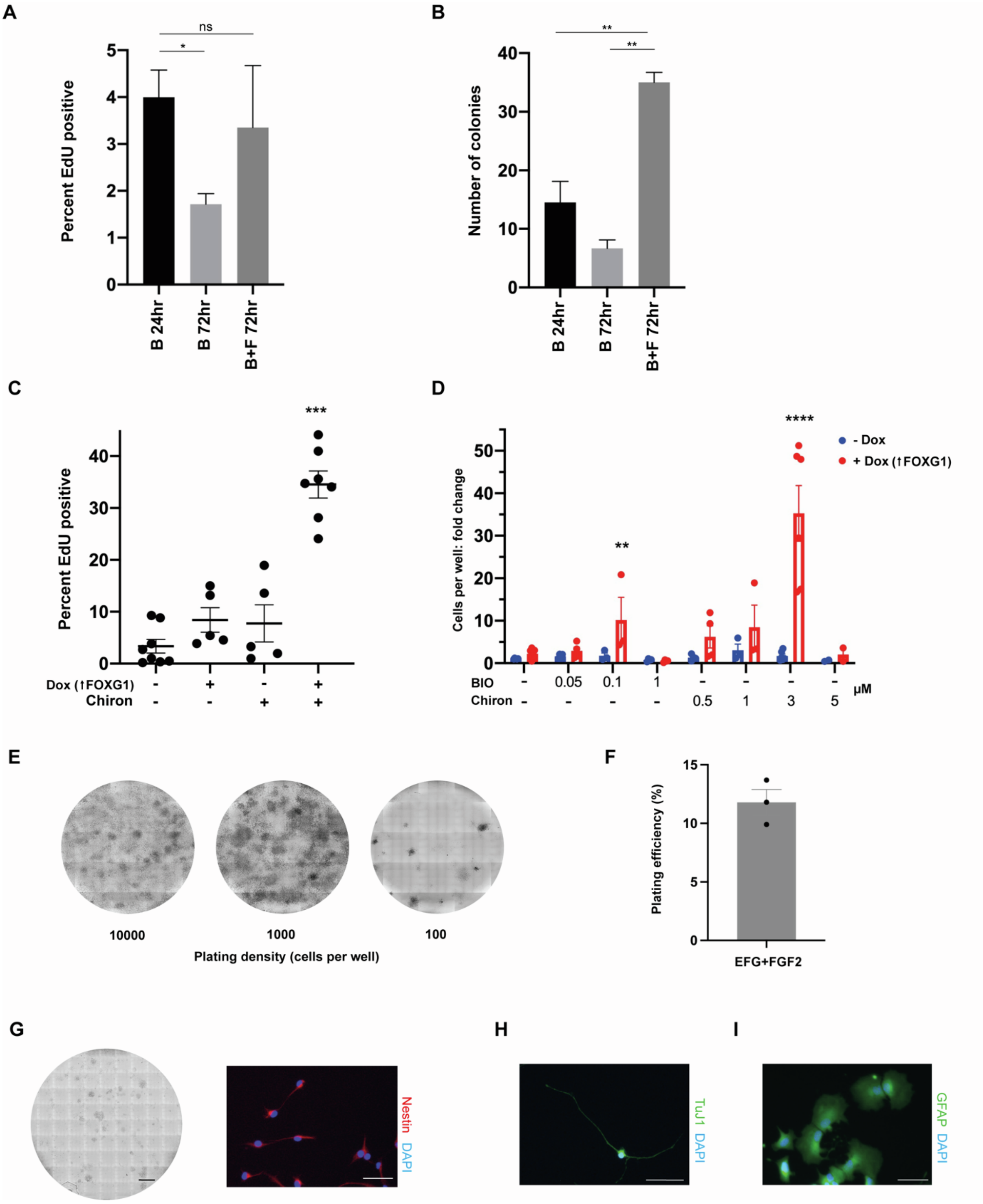

Figure S2. Confirmation of synergy between FOXG1 and GSK3 inhibition. Related to Figure 2. (A) Quantification of EdU incorporation in F6 after exposure to BMP4 for 24 hr (B24hr) or 72 hr (B72hr) or to BMP4+FGF2 for 72hr (B+F72hr) then to EGF+FGF2 for 4 days, showing minimal EdU incorporation in all conditions. One-way ANOVA with Tukey's multiple comparison tests. Performed in triplicate. One-way ANOVA with Tukey's multiple comparison tests. (B) Quantification of colony formation in F6 after exposure to BMP4 for 24 hr (B24hr) or 72 hr (B72hr) or to BMP4+FGF2 for 72hr

(B+F72hr) then to EGF+FGF2 for 10 days. Means of 3-6 replicates. One-way ANOVA with Tukey's multiple comparison tests. (C) EdU incorporation after 72hr BMP4+FGF2 and return to EGF+FGF2 +/- Dox and/or Chiron for 4-6 days, comparable to the equivalent assay with 24hr BMP4 exposure (Figure 2B). n=8 independent replicates; 15 technical replicates each. Kruskal-Wallis test with Dunn's multiple comparison tests. Significance for comparison to EGF+FGF2 shown. (D) Increase in F6 cell number after BMP4 exposure for 24hr and return to EGF+FGF2 alone or with varying doses of BIO or Chiron +/- Dox for 6 days. Two-way ANOVA. Significance in comparison to EGF+FGF2 alone shown. n=6 independent replicates; >3 technical replicates each. (E) Representative images of serial dilution colony assays: 10000, 1000, 100 cells plated per well (6 well plate) in BMP4 with return to EGF+FGF2+Dox+Chiron for 10 days. Scale bars 2mm. (F) Quantification of colony forming efficiency (% of cells plated which give rise to colonies) for F6 cells plated in EGF+FGF2 and never exposed to BMP4. n=3. (G) Representative image of colony formation from serially passaged F6 cells following Dox+Chiron exposure. Scale bar 1cm. Representative image of Nestin expression in cells in this assay. Scale bar 50μm. DAPI (blue), Nestin (red). Cells were plated at 100 cells/well in 6 well plates in BMP4 media, then changed to EGF+FGF2 + Dox + Chiron after 24 hours. After 8 days, colonies had formed and these were picked and replated in EGF+FGF2 in 6 well plates at low density. After 2 weeks, colonies had formed and these were replated in EGF+FGF2 media in 10cm dishes at low density. After 2 weeks, plates were fixed and either stained with methylene blue and imaged on the Celigo Image Cytometer (Nexcelom) or subjected to immunocytochemistry for Nestin. (G&H) Representative images of F6 cells. Cells were plated at 100 cells/well in 6 well plates in BMP4 media, then changed to EGF+FGF2 + Dox + Chiron after 24 hours. After 8 days, colonies had formed. Media was changed to either 10% fetal calf serum for 5 days (for astrocyte differentiation assay, G) or FGF2 media (withdrawal of EGF) for 24 hours, then media without growth factors for 7 days (for neuronal differentiation assay, H). Scale bars 50μm.

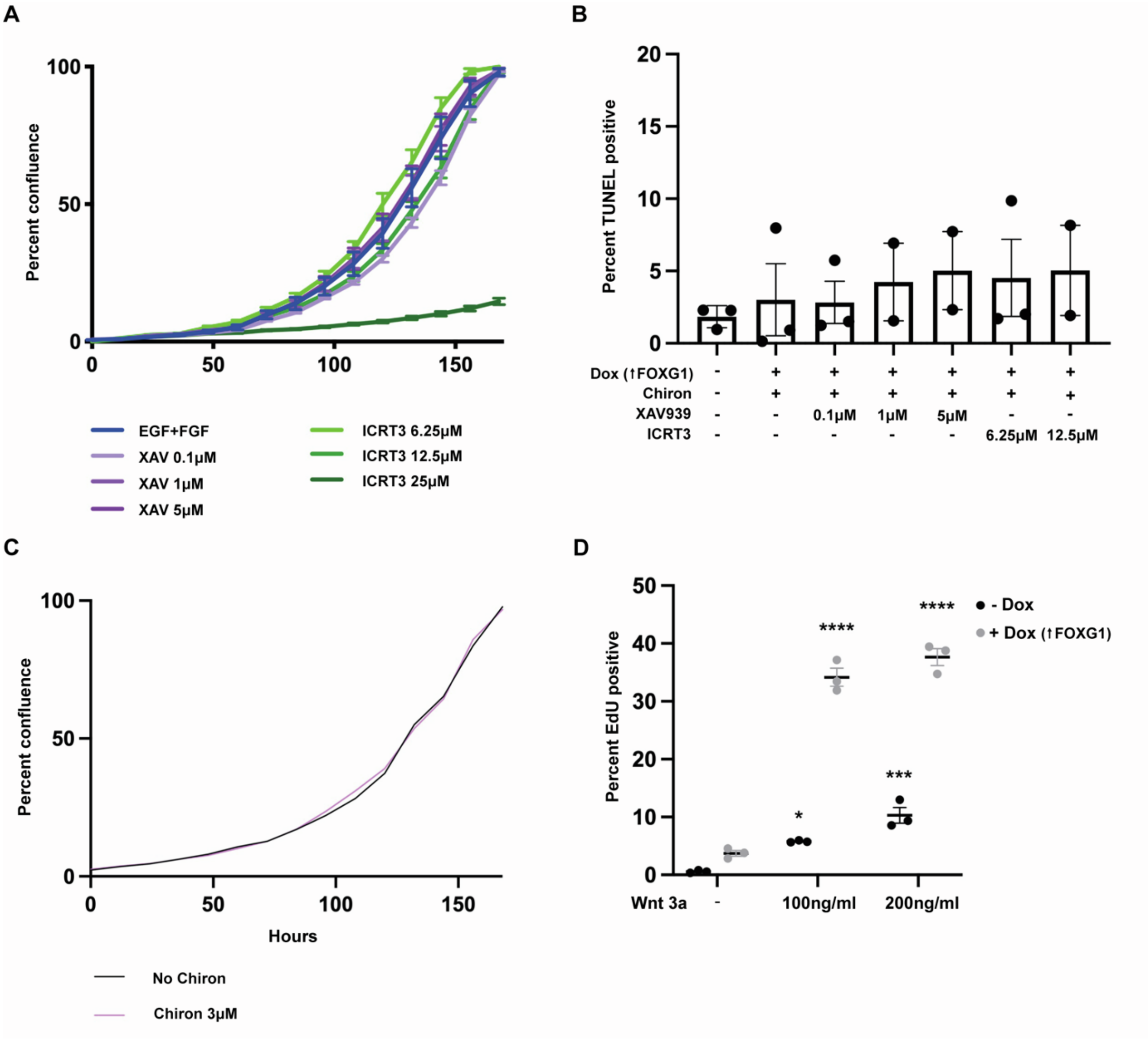

Figure S3. Wnt activation and inhibition confirm Wnt pathway involvement in the synergy with FOXG1 on quiescence exit; Wnt activation is not essential for proliferation. Related to Figure 3. (A) Growth curves showing that XAV939 has no effect on the proliferation of NSCs (F6) in EGF+FGF2 up to a concentration of 5μM and that ICRT3 has no effect on proliferation up to a dose of 12.5μM. n=6. (B) TUNEL assay confirming no significant increase in apoptosis in F6 cells with Wnt inhibitors as compared to EGF+FGF2 alone. One-way ANOVA. Non-significant. Performed in independent duplicate. (C) Growth curve of F6 cells plated in EGF+FGF2 +/- Chiron. (D) Quantification of EdU incorporation in F6 cells after BMP4+FGF2 72hr and return to EGF+FGF2 +/- Wnt3a +/- Dox for 4 days (at this timepoint, cell number changes were minimal). Two-way ANOVA. Performed in independent triplicate; 6 technical replicates each.

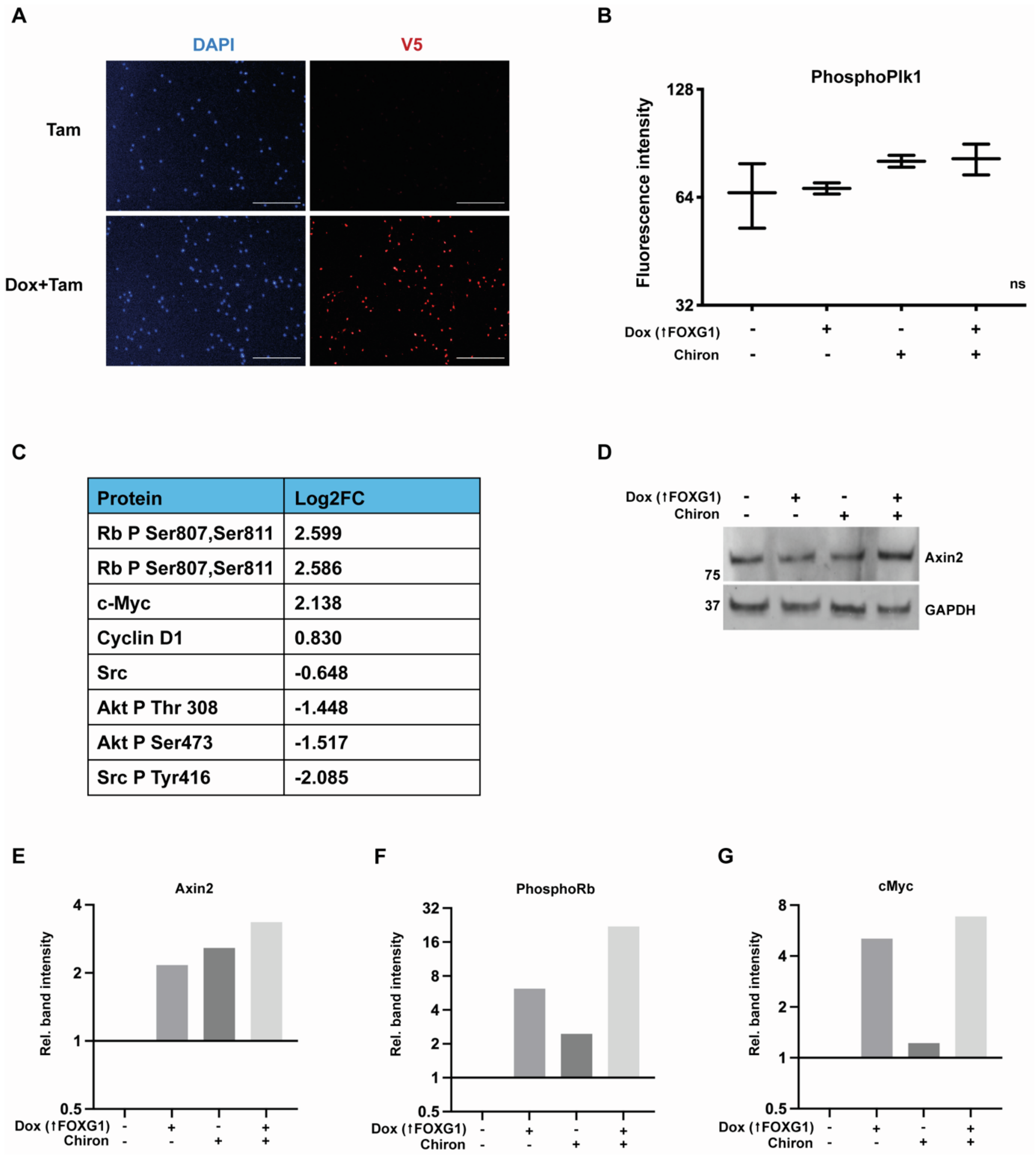

Protein	Log2FC
Rb P Ser807,Ser811	2.599
Rb P Ser807,Ser811	2.586
c-Myc	2.138
Cyclin D1	0.830
Src	-0.648
Akt P Thr 308	-1.448
Akt P Ser473	-1.517
Src P Tyr416	-2.085

Figure S4. Additional data pertaining to RPPA and to the F6BC1 line. Related to Figure 4. (A) V5 staining confirms that the inducible FOXG1 cassette is intact in the F6BC1 cell line and that it is not activated by tamoxifen. DAPI (blue), V5 (red). Scale bars 150μm. (B) Relative levels of mitotic marker phosphoPlk1 showing no significant difference between the 4 conditions in the RPPA assay. Kruskal-Wallis test. Data plotted are medians of 4 serially diluted dots from 3 technical replicates of independent duplicates. (C) Table of all significant hits (T-tests with Holm-Sidak correction, cut off p value <0.05) from RPPA with Log2 fold change in fluorescence intensity between EGF+FGF2 samples and EGF+FGF2+Dox+Chiron samples. (D) Western blot for Axin2 in F6 cells exposed to BMP4 for 24hr and

then to EGF+FGF2 +/- Dox and/or Chiron for 2 days. (E) Quantification of band intensity for the blot shown in panel D, normalized to GAPDH. Quantified with ImageJ software. (F&G) Quantification of band signal for the Western blot shown in Figure 4G. Quantified with ImageJ software.

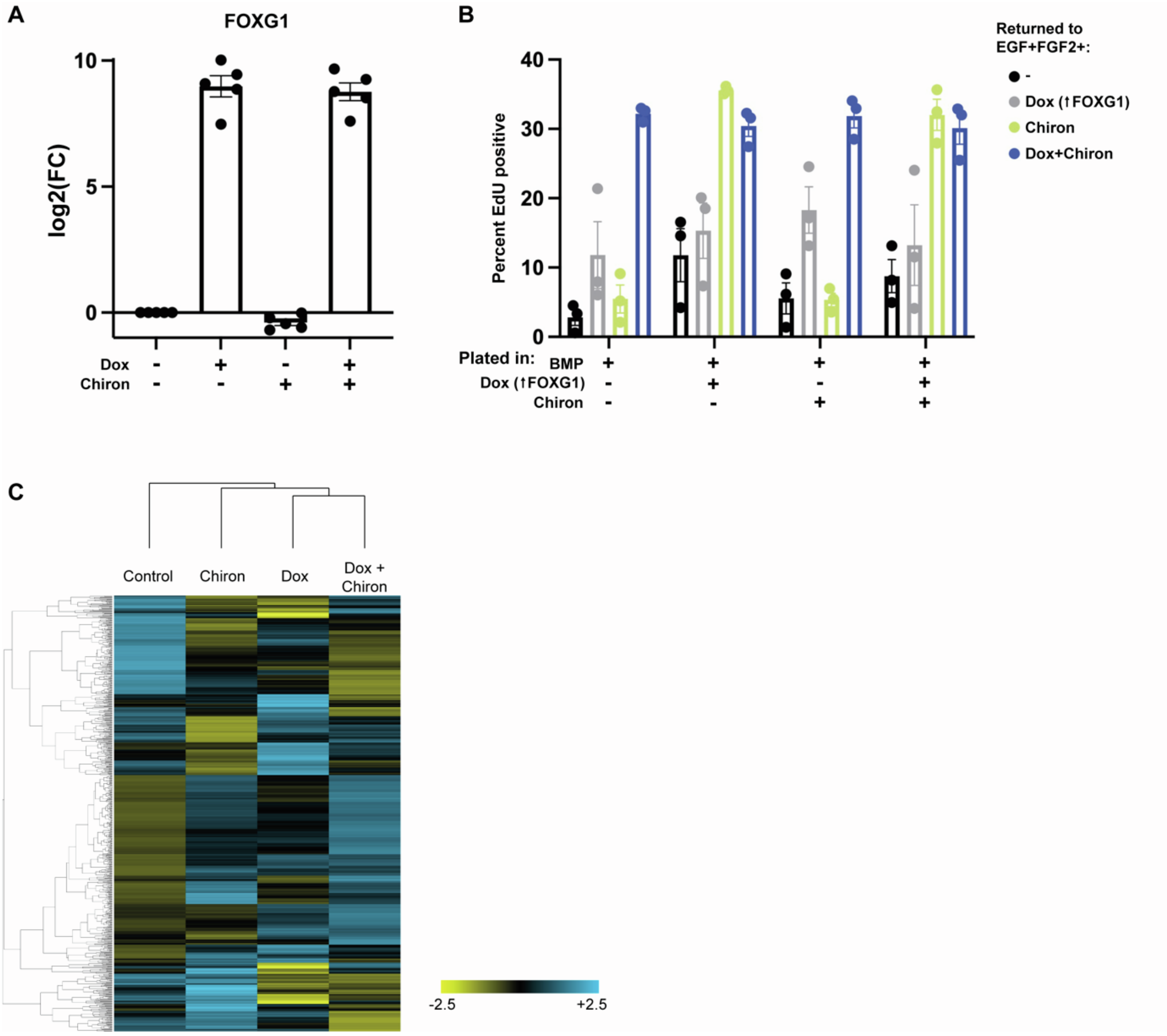

Figure S5. FOXG1 primes cells to respond to GSK3 inhibition; additional data pertaining to Nanostring in F6. Related to Figure 5. (A) qRT-PCR in F6 cells after 72hr in BMP4+FGF2 then 72hr in EGF+FGF2 +/- Dox and/or Chiron shows that human FOXG1 is elevated to similar levels in Dox and Dox+Chiron, as expected. n=5 independent replicates; 3 technical replicates each. (B) EdU incorporation in F6 cells after exposure to BMP4 +/- Dox and/or Chiron shows that Dox (induction of FOXG1 overexpression) during BMP4 exposure primes cells to exit quiescence in response to Chiron, resulting in similar exit from quiescence, in primed cells, to Dox+Chiron. n=3 independent replicates; 3 technical replicates each. (C) Heatmap of NanoString data.

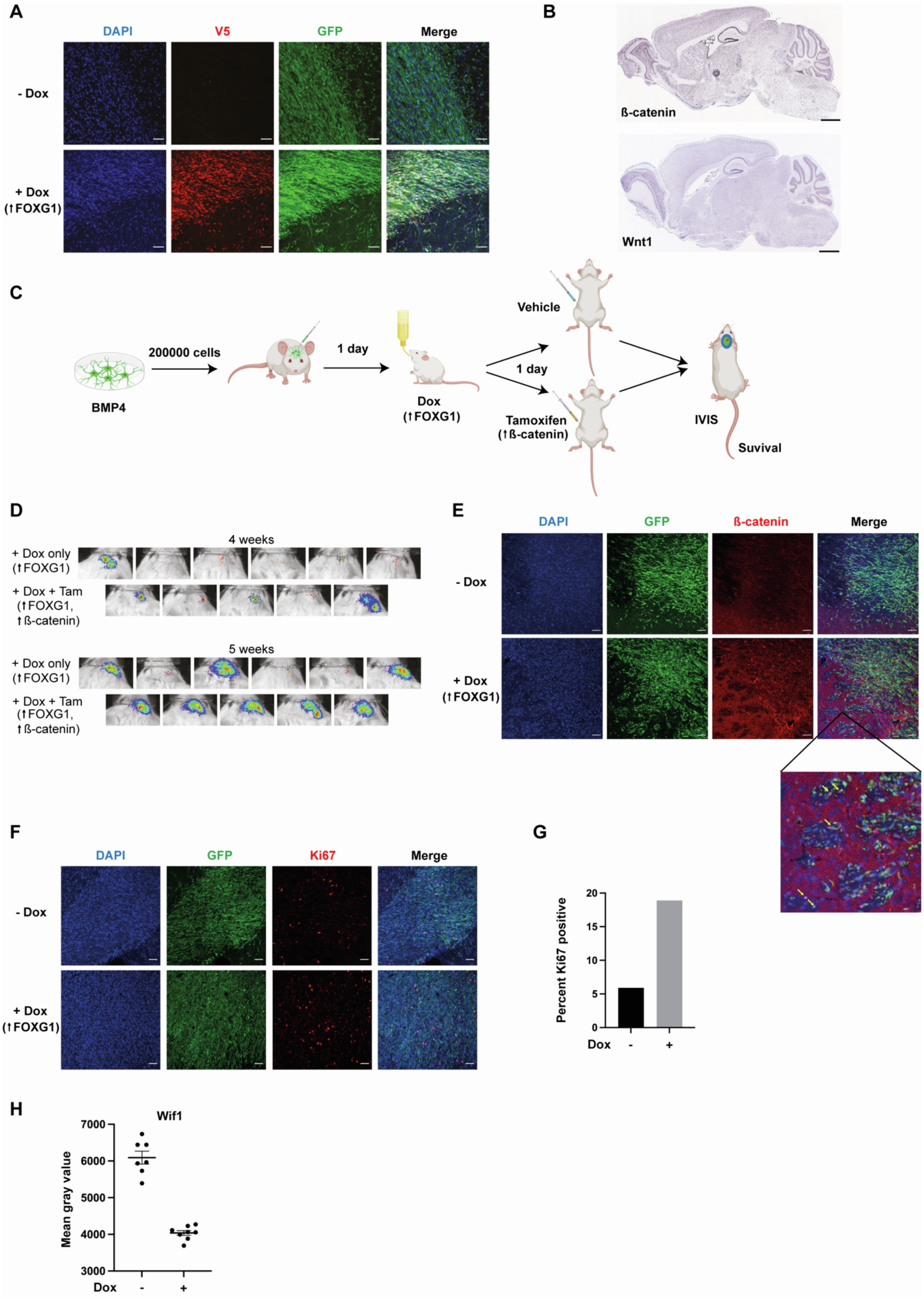

Figure S6. Additional data pertaining to in vivo findings. Related to Figure 6. (A) Representative images showing GFP positive tumour cells generated from F6BC1NPE cells, showing expression of V5

in the context of Dox administration. DAPI (blue), V5 (red), GFP (green). Scale bars 50μm. (B) ISH for Ctnnb1 and Wnt1 in the adult mouse brain, mouse.brain-map.org. Scale bar 1250μm. (C) Schematic of the experiment used to evaluate the impact of tamoxifen on tumour growth in this model. Created with BioRender. (D) IVIS images at 4 and 5 weeks for mice (cohort 1 of 2) given Dox in drinking water +/- IP injection of Tamoxifen. (E) Representative images showing increased ß-catenin expression in the context of Dox administration in F6BC1NPE tumours in mice culled at 21 days. DAPI (blue), ß-catenin (red), GFP (green), scale bars 50μm. Expanded image shows some cells expressing nuclear ß-catenin (arrows). (F) Representative images showing increased Ki67 expression in the context of Dox administration in F6BC1NPE tumours in mice culled at 21 days. DAPI (blue), Ki67 (red), GFP (green). Scale bars 50μm. (G) Quantification of Ki67 positivity in these tumours. (H) Quantification of WIF1 signal intensity in F6BC1NPE tumours in mice given Dox or no Dox and culled at 21 days n=8.

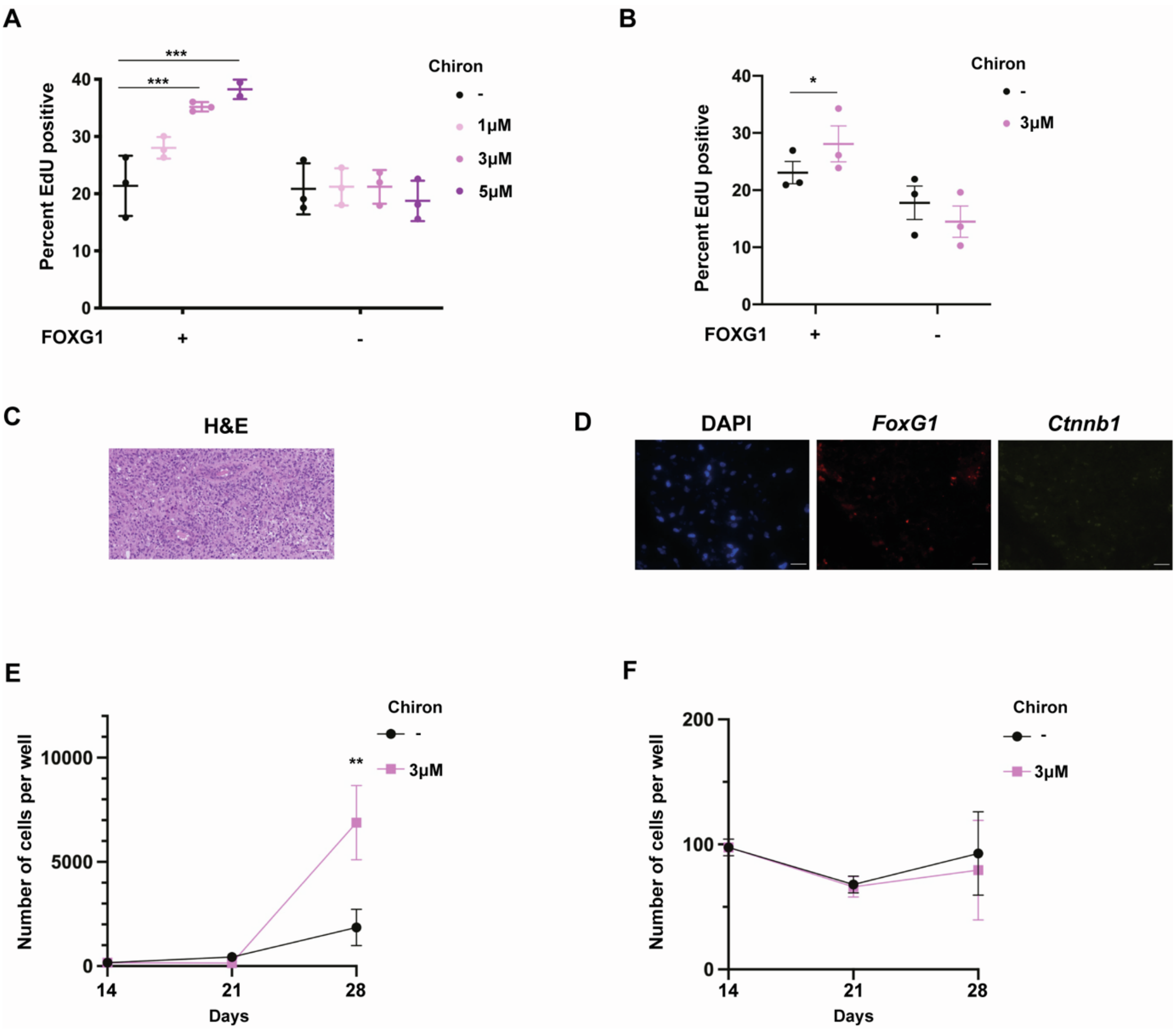

Figure S7. Additional data pertaining to the findings in human GSCs. Related to Figure 7. (A&B) Quantification of EdU incorporation in (A) G7 and G7 FOXG1 KO cells and in (B) GBM002 and GBM002 KO cells, showing that Chiron drives exit from quiescence in a dose-dependent manner, only in the context of intact FOXG1. Two-way ANOVA with Sidak's multiple comparison tests. n=3 independent replicates; 15 technical replicates each. (C) Haematoxylin and eosin stain of patient GBM sample G313. Scale bar 100 μm (D) Representative images of RNAScope peformed on G313 with negative control probes. DAPI (blue), *FOXG1* mRNA (red); *CTNNB1* (ß-catenin) mRNA (yellow). Scale bars 50 μm. (E&F) Cell number per well in (E) GBM002 and (F) GBM002 FOXG1KO cells plated at 1000 cells/well at day -1 and irradiated at day 0 with 8Gy. Chiron or DMSO control added to media at day 14, n=6-12. Linear regression analysis.

Mean +Dox	Mean -Dox	SD +Dox	SD -Dox	Z’
10477.27	1729.733	2629.409	435.2303	0.246832
8803.5	1858.767	2153.094	355.0453	0.294295
2021.067	717.6	452.8303	85.91881	0.409842
1969.967	726.9333	310.7397	61.65193	0.585756

Table S1. Z’ for 4 plates of FOD3 cells during screen optimisation. Each row represents a plate seeded using successive techniques and the Multidrop Combi reagent dispenser (ThermoFisher).

Gene mRNA	Log2FC	Gene mRNA	Log2FC	Gene mRNA	Log2FC	Gene mRNA	Log2FC	Gene mRNA	Log2FC	Gene mRNA	Log2FC	Gene mRNA	Log2FC
Wif1	-4.23	Map2k1	-1.49	Acvr2a	-1.05	Sos2	-0.716	Acvr1b	-0.48	Atr	0.543	Rfc4	1.27
Map3k5	-3.57	Cacnb2	-1.47	Id1	-1.04	Tiam1	-0.715	Aph1b	-0.473	Rad50	0.656	Ets2	1.35
Hmga2	-2.97	Bdnf	-1.45	Ppp3ca	-1.03	Apc	-0.71	Smo	-0.472	Col5a1	0.69	Mdc1	1.38
Etv4	-2.85	Kit	-1.44	Fgfr1	-1.03	Id2	-0.705	Stag2	-0.463	Casp7	0.694	Pole2	1.39
Fos	-2.58	Mapk10	-1.42	Smad9	-1.03	Pold4	-0.701	Them4	-0.455	Npm1	0.713	Cntfr	1.4
Fn1	-2.43	Zic2	-1.39	Prkaa2	-1.03	Crlf2	-0.68	Rbx1	-0.446	Tnfrsf10b	0.744	Nasp	1.6
Il1r1	-2.34	Igf1r	-1.38	Smad3	-1.02	Capn2	-0.677	Stk11	-0.444	Fancf	0.82	Chek2	1.73
Gas1	-2.34	Gnaq	-1.38	Idh1	-1.01	Kdm6a	-0.675	Spop	-0.438	Cdkn2d	0.849	Efna2	1.81
Gpc4	-2.33	Lef1	-1.37	Pbx3	-1.01	Abl1	-0.659	Fbxw11	-0.437	Hmga1	0.857	H2afx	1.84
Pdgfc	-2.29	Itga6	-1.36	Pik3ca	-0.995	Mapk8ip1	-0.65	Ppp3r1	-0.429	Shc4	0.893	E2f1	1.87
Dusp6	-2.26	Mllt3	-1.3	Rras2	-0.993	Pld1	-0.65	Mapk3	-0.416	Ezh2	0.893	Brca1	2.22
Spry1	-2.17	Gadd45g	-1.3	Prkar2a	-0.982	Idh2	-0.641	Smad4	-0.407	Rac3	0.911	Mcm7	2.22
Ptch1	-2.1	Tnfaip3	-1.3	Nfkbia	-0.964	Wnt5b	-0.638	Kras	-0.407	Cdkn1a	0.927	Pold1	2.28
Nog	-2.1	Skp1a	-1.3	Ppp2cb	-0.963	Irs1	-0.631	Ikbkb	-0.403	Cacnb3	0.961	Rad51	2.3
Kitl	-2.06	Prkca	-1.3	Bcl2l1	-0.948	Rac1	-0.63	Chuk	-0.374	Blm	0.962	Ccne1	2.42
Fgf12	-2.06	Cdkn1b	-1.28	Lifr	-0.947	Braf	-0.627	Bap1	-0.373	Myc	0.974	Hist2h3b	2.46
Nfkbiz	-2.03	Ptk2	-1.27	Egfr	-0.943	Nfatc1	-0.623	Prkaca	-0.372	Fen1	0.981	Hist1h3b	2.47
Spry	-2.02	Irak2	-1.26	Jak2	-0.939	Mapk9	-0.62	Sos1	-0.364	Wee1	0.982	Hells	2.6
Casp12	-2.01	Gadd45a	-1.23	Hspa2	-0.938	Endog	-0.617	Grb2	-0.362	Tgfb3	0.985	Ube2t	2.65
Gng12	-2.01	Wnt5a	-1.22	Xrcc4	-0.932	Stat1	-0.611	Ppp2r1a	-0.353	Cacna1h	0.986	Nkd1	2.74
Fgf1	-1.96	Zbtb16	-1.22	Fzd3	-0.911	Jak1	-0.598	Smarcb1	-0.341	Hes1	0.989	Mcm2	2.75
Itgb8	-1.94	Igfbp3	-1.2	Nfkb1	-0.901	Stat3	-0.598	Mtor	-0.34	Cdc25a	0.989	Stmn1	2.82
Six1	-1.82	Casp3	-1.18	Mllt4	-0.887	Nfe2l2	-0.59	Arnt2	-0.327	Atm	1.04	Socs2	2.86
Plcb4	-1.79	Gnas	-1.15	Nf2	-0.883	Xpa	-0.586	Ifnar1	-0.319	Cdk2	1.05	Chek1	2.91
Tspan7	-1.79	Insr	-1.14	Tcf7l1	-0.882	Rhoa	-0.576	Mapk1	-0.293	Efna3	1.07	Ccne2	3.15
Ppargc1a	-1.78	Map3k1	-1.14	Etv1	-0.849	Map2k4	-0.575	Alkbh3	-0.27	Skp2	1.08	Top2a	3.27
Cacnb4	-1.77	Ifnar2	-1.12	Mnat1	-0.841	Dvl3	-0.571	Map3k7	-0.24	Bmp7	1.1	Mcm5	3.32
Hist2h3c2	-1.76	Cyld	-1.11	Camk2b	-0.812	Hdac4	-0.569	Smarca4	0.157	Axin2	1.14	Ttk	3.68
Cd14	-1.71	Angpt2	-1.11	Cdkn2c	-0.791	Cul1	-0.557	Fubp1	0.352	Suv39h2	1.2	Il6ra	3.77
Fut8	-1.61	Shc3	-1.09	Smad1	-0.774	Ccnd2	-0.557	Cdk4	0.472	Dnmt1	1.21	Ccna2	4.03
Hspa1a	-1.59	Fzd7	-1.09	Maml2	-0.75	Mlh1	-0.556	Tfdp1	0.519	Cdc7	1.21	Cdc25c	4.32
Ikbkg	-1.51	Tlr4	-1.09	Prkacb	-0.742	Foxo4	-0.504	Rfc3	0.527	Pcna	1.23		
Bambi	-1.5	Map3k8	-1.08	Hsp90b1	-0.717	Rpa3	-0.497	Dnmt3a	0.539	Mcm4	1.24		

Table S2. Table of all results from NanoString with Log2 fold change in count between EGF+FGF2 samples and EGF+FGF2+Dox+Chiron samples. Statistics using NSolver Advanced Analysis software.

Trp53_L, sgRNA	Gangoso et al[40], Integrated DNA Technologies	GCTGGCAGAATAGCTTATTG
Trp53_R, sgRNA	Gangoso et al[40], Integrated DNA Technologies	GAGCGCAAAGAGAGGTACGC
Pten_L, sgRNA	Gangoso et al[40], Integrated DNA Technologies	GGTTTGATAAGTTCTAGCTG
Pten_R, sgRNA	Gangoso et al[40], Integrated DNA Technologies	GTAAATACGTTCTTCATACC
Nf1_L, sgRNA	Gangoso et al[40], Integrated DNA Technologies	TCATCATCACATCTTCGGAT
Nf1_R, sgRNA	Gangoso et al[40], Integrated DNA Technologies	TCGGCTGCTTTGGAACAATC
hEGFRvIII_Fwd primer	Gangoso et al[40], Sigma	ATCACAAGTTTGTACAATGCGACCCTCCGGGACGGCC
hEGFRvIII_Rev primer	Gangoso et al[40], Sigma	CACCACTTTGTACATCATGCTCCAATAAATTCACT
3FLAG-LUC-2AGFP Fwd primer	Gangoso et al[40], Sigma	GGGGACAAGTTTGTACAAAAAAGCAGGCTTCGCCACCATGGACTACAAAGA
3FLAG-LUC-2AGFP Rev	Gangoso et al[40], Sigma	GGGGACCACTTTGTACAAGAAAGCTGGGTTTTACTTGTACAGCTCGTCCA
FOXG1 sgRNA-1	Bulstrode et al[19], Sigma	CCGCCCTGGACGGGGCTAA
FOXG1 sgRNA-2	Bulstrode et al[19], Sigma	GCAAGGGCGAGCCGGGCGG
Foxo3_gRNA1	Bulstrode et al[19], Sigma	CGCGTTCAGAATGAAGGCACGGG
Foxo3_gRNA2	Bulstrode et al[19], Sigma	CGCATGAAGCGGCTGTGCAGGG

Table S3. Oligonucleotides.
